# Ge–Sb–S–Se–Te amorphous chalcogenide thin films towards on-chip nonlinear photonic devices

**DOI:** 10.1038/s41598-020-67377-9

**Published:** 2020-07-17

**Authors:** J.-B. Dory, C. Castro-Chavarria, A. Verdy, J.-B. Jager, M. Bernard, C. Sabbione, M. Tessaire, J.-M. Fédéli, A. Coillet, B. Cluzel, P. Noé

**Affiliations:** 1 Université Grenoble Alpes, CEA, LETI, MINATEC Campus, 17 Avenue des Martyrs, 38000 Grenoble, France; 2 Université Grenoble Alpes, CEA, IRIG, MINATEC Campus, 17 Avenue des Martyrs, 38000 Grenoble, France; 3 ICB, UMR CNRS 6303, Université de Bourgogne Franche Comté, 9, Avenue Alain-Savary, BP 47870, 21078 Dijon cedex, France

**Keywords:** Nonlinear optics, Optical materials and structures

## Abstract

Thanks to their unique optical properties Ge–Sb–S–Se–Te amorphous chalcogenide materials and compounds offer tremendous opportunities of applications, in particular in near and mid-infrared range. This spectral range is for instance of high interest for photonics or optical sensors. Using co-sputtering technique of chalcogenide compound targets in a 200 mm industrial deposition tool, we show how by modifying the amorphous structure of GeSb_w_S_x_Se_y_Te_z_ chalcogenide thin films one can significantly tailor their linear and nonlinear optical properties. Modelling of spectroscopic ellipsometry data collected on the as-deposited chalcogenide thin films is used to evaluate their linear and nonlinear properties. Moreover, Raman and Fourier-transform infrared spectroscopies permitted to get a description of their amorphous structure. For the purpose of applications, their thermal stability upon annealing is also evaluated. We demonstrate that depending on the GeSb_w_S_x_Se_y_Te_z_ film composition a trade-off between a high transparency in near- or mid-infrared ranges, strong nonlinearity and good thermal stability can be found in order to use such materials for applications compatible with the standard CMOS integration processes of microelectronics and photonics.

## Introduction

Chalcogenides are commonly defined as non-oxide compounds containing at least one chalcogen element such as S, Se and/or Te (belonging to group 16 of O) alloyed with electropositive elements (more often elements of group 15 (As, Sb, Bi) and/or group 14 (Si, Ge, Sn, Pb)). Chalcogenide exhibit a unique portfolio of properties which has led to their wide use for non-volatile memory applications such as optical data storage (CD-RW and DVD-RAM), Conductive-Bridging Random Access Memory or Phase-Change Random Access Memory^1^. More recently, thanks to the huge electronic nonlinearities and discontinuity [in particular the Ovonic Threshold Switching (OTS) mechanism] observed in some chalcogenide glasses (CGs) under electrical field application, the latter are considered as the most promising materials to be used as innovative selector element in 3D memory arrays^2,3^. Besides, thanks to a high transparency window in the infrared range and large optical nonlinearities^4,5^, chalcogenide glasses offer also opportunities for elaboration of innovative mid-infrared (MIR) components such as MIR super-continuum (SC) laser sources, optical sensors, IR micro-lens arrays and all-optical integrated circuits^6,7^. The potential of amorphous semiconductors for optical applications is already demonstrated but the progresses in glass science is still behind crystal science due to inherent complexity of highly disordered systems hindering thus their structural description and theoretical modelling^8^. Up to now, state-of-the-art of MIR SC generation have been achieved by using mainly chalcogenide compounds containing Arsenic such as As_2_S_3_ and As_2_Se_3_ fibres^9, 10^ or GeAsSe rib waveguides^11^. However, the R.E.A.C.H. European recommendation (https://echa.europa.eu/regulations/reach) as well as a recent publication from the World Health Organization (https://www.who.int/ipcs/assessment/public_health/chemicals_phc/en/) have both identified Arsenic as one of the ten most harmful chemicals for human health. As a result, developing new materials using less and less harmful or rare element is a huge challenge for the materials science.

In that context, the aim of the present study is to develop As-free amorphous chalcogenide thin films compatible with CMOS technology of microelectronics and photonics. The ultimate purpose would be to give clear clues to select the most suitable chalcogenide compositions exhibiting the best trade-off between stability and optical properties in order to enable future achievement of passive and active on-chip MIR components such as MIR optical waveguides or portable SC laser sources. To this aim, a wide composition range of amorphous GeSb_w_S_x_Se_y_Te_z_ chalcogenide thin films were deposited on 200 mm Si-based substrates by means of magnetron co-sputtering technique. Despite the ability of such technique to permit a fast and easy study of a wide range of chalcogenide thin films’ compositions, only very few studies of the optical properties of co-sputtered chalcogenide films have been reported in literature yet^12–14^. Since the local structure of glasses determines their physical properties, a first description of the amorphous structure of the (co-)sputtered chalcogenide films was probed by means of Raman and Fourier-transform infrared (FTIR) spectroscopies. Then, modelling of the spectroscopic ellipsometry data acquired on as-deposited thin films permits to determine their linear optical constants in the visible to near infrared range. From these experimental data and by using the well-known Sheik–Bahae model, a first evaluation of their n_2_ Kerr refractive index is given in order to get better insight on their nonlinear optical properties. Moreover, the thermal stability of all deposited thin films was probed by monitoring their optical reflectivity during annealing under N_2_ atmosphere. Finally, some of the GeSb_w_S_x_Se_y_Te_z_ compositions are shown to exhibit an optimized compromise between the good glass stability (but limited transparency window) of S-based chalcogenide and the high 3rd order nonlinear refractive index n_2_ (but low thermal stability of the amorphous phase) of Te-based compositions^15,16^. Besides, we reveal the major impact of Sb introduction in GeS_x_ and GeSe_x_ binary as well as GeS_x_Se_y_ and GeSe_x_Te_y_ ternary compounds on the amorphous structure and therefore on the optical properties of chalcogenide thin films obtained by co-sputtering deposition technique.

## Methods

### Chalcogenide thin films deposition and basic characterization

The amorphous chalcogenide thin films were obtained by means of (co-)sputtering on 200 mm Si(001) substrates from either a single or up to simultaneously three targets of pure chalcogenide compounds. Before (co-)sputtering deposition, we did not perform any particular surface preparation of the Si substrates. Indeed, the substrates are standard high-quality microelectronic Si wafers with high purity and very low surface contamination and covered, as usual, by a few nm thick thin layer of native silicon oxide. The films’ thicknesses were either fixed to 100 nm or 200 nm. The compositions of sputtering targets were Ge_40_S_60_, Ge_37_S_63_, Ge_26_Se_74_, Ge_30_Se_70_, Ge_52_Te_48_, pure Sb and pure Ge. The targets were sputtered by magnetron sputtering with an Ar plasma either achieved by using radio frequency (RF) at 13.56 MHz or direct courant (DC) plasma discharges. It is well known that deposition parameters of magnetron sputtering technique, such as Argon gas flow, substrate temperature, deposition vessel geometry, RF or DC plasma, applied power on targets and deposition pressure especially can play a major role on the density, composition and structure of the thin films^17–19^. In this study, all deposition were made using the same Ar flow and the deposition pressure was kept at 5.10^–3^ mBar. Note that due to geometry and deposition conditions in our multi-cathode chamber, the substrate temperature was kept very close to room temperature during deposition of chalcogenide films.

The different compositions of the sputtered films were obtained by varying the power applied to the targets between 10 and 220 W (see Sect. 1 of the Supplementary Information). Note that a slight composition deviation between targets and deposited films can occur. This is the result of the different sputtering yields between chemical elements due to a selective atoms-ions interactions. This effect also depends on the deposition conditions such as applied power or aging of the targets. For instance, Ge_38_S_62_ and Ge_36_S_64_ thin film samples were obtained by means of sputtering of the same Ge_37_S_63_ target and in the same deposition conditions. The slight deviation of composition is probably due to aging of the target after a high number of deposition cycles. This variation is of the order of the accuracy level of characterization methods used to determine films' compositions (~ 1 at. %). However, this trend on compositions is also confirmed by changes of properties and amorphous structure of these films as shown in the following.

The different compositions of the films were adjusted by varying the deposition rates ratio in between the different sputtering targets during the co-sputtering deposition. Then, the obtained compositions were verified by means of Wavelength Dispersive X-Ray Fluorescence (WDXRF) and/or ion beam analysis (Rutherford Back Scattering (RBS) and Particle Induced X-Ray Emission (PIXE) measurements).

Immediately after deposition all films were transferred under high vacuum in a second deposition chamber in order to be capped by a 10 nm thick SiN_x_ protective layer. This nitride layer deposited by reactive RF magnetron sputtering of a pure Si target under Ar/N_2_ reactive atmosphere is shown to be highly efficient to protect chalcogenide thin films from surface oxidation since chalcogenides are particularly prone to oxidation^18,20,21^. We must note that during SiN_x_ deposition the temperature of the chalcogenide thin film samples can reach up to 180 °C due to thermal heating resulting from RF sputtering of the Si target. We note that since this temperature is below the glass transition temperature of our chalcogenide samples, it is expected to have no detrimental impact on the films, as already studied for similar chalcogenide bulk glasses and thin films^4,22–27^.

In order to get a first evaluation of the limit of stability of the deposited amorphous chalcogenides upon annealing, the reflectivity of the films at 670 nm was monitored during an annealing under N_2_ atmosphere. The heating ramp rate was fixed to 10 °C/min. The limit temperature was defined as the temperature for which an irreversible change of sample reflectivity was detected indicating an irreversible structural modification of the amorphous material (phase crystallization, phase segregation, layer delamination …).

### FTIR and Raman scattering measurements

Fourier-Transform Infrared spectroscopy (FTIR) analysis of local order of the amorphous films was performed in transmission mode in the 100–500 cm^−1^ range. All absorbance spectra were acquired in the same experimental conditions (average over 32 scans and spectral resolution of 2 cm^−1^). A reference absorbance spectrum collected on a Si(100) substrate covered by a 10 nm SiN_x_ capping layer was used as background subtraction for all FTIR spectra acquired on the chalcogenide thin film samples. Then, all raw spectra were normalized to the chalcogenide film thicknesses in order to get a more accurate comparison between the different thin film samples.

Raman scattering spectra were acquired in a micro-Raman spectrometer in the range from 100 to 500 cm^−1^ using a laser probe at 532 nm wavelength. The acquisition conditions (laser power, magnification and exposure time) were adjusted for each films in order to optimize the signal-to-noise ratio but with a particular emphasis to keep no or very limited impact on the material’s structure.

### Spectroscopic ellipsometry measurements

Spectroscopic ellipsometry (SE) measurements were performed in the 400–1,700 nm range. Data were collected at three incidence angles (55, 65 and 75°). Analysis of the raw data was performed using WVASE 32^®^ software. A 10 nm SiN_x_ layer deposited on a Si substrate was also measured separately in order to take into account any possible influence of capping layer when modelling of chalcogenide films’ data. For chalcogenide thin film samples, the film thicknesses, dielectric functions, optical constants (refractive index n and extinction coefficient k) and absorption coefficient α as a function of the photon energy in the 0.73–3.1 eV range were obtained by means of modelling of the SE data with a Cody-Lorentz (CL) model (see also Sect. 3 of the Supplementary Information).

The optical bandgaps of the films were estimated by using the bandgap values obtained from the CL fitting model (E_g_^CL^) as well as by considering the energy for which the absorption reaches 10^4^ cm^-1^ (E_g_^04^).

Using the M-line technique^28^ at two wavelengths (1,313 and 1,548 nm), the effective refractive indices of the films as well as their thicknesses (by prism coupling technic) were also accurately determined (not shown). The obtained values of n at 1,313 and 1,548 nm were compared with those obtained from SE modelling and were used to validate the accuracy of SE results.

In order to get an estimation of the optical nonlinearities of the studied glasses the well-known Sheik–Bahae model was used^29^. This method allows to estimate nonlinear Kerr refractive index n_2_ by means of an analytical approach using linear refractive index and optical band gap energy values. This model takes into account contributions from several physical origins: two-photon and Raman transitions, linear Stark and quadratic Stark effects. A divergent term is also added in order to subtract the unphysical behaviour resulting from the formula used to adjust these contributions. For these calculations, we used a Sellmeier fit of the refractive index in the material’s transparency range obtained by using the Cody-Lorentz modelling and the optical band gap energy E_g_^04^. The results of Sellmeier fits were extrapolated to wavelengths beyond spectral range of the ellipsometry measurements.

## Results and discussion

### As-deposited chalcogenide thin films’ composition map

**Figure 1:**
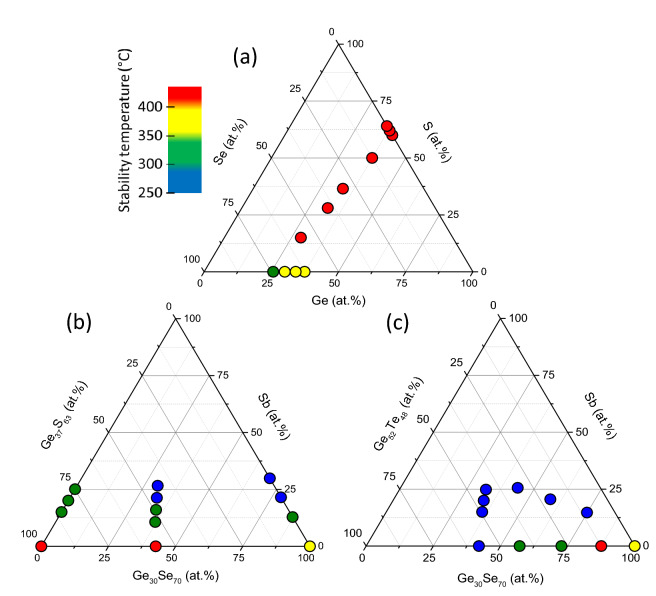
Ternary and pseudo-ternary diagrams showing compositions of the chalcogenide thin films deposited by magnetron (co)-sputtering: (**a**) Ge_1-x-y_S_x_Se_y_, (**b**) [Ge_37_S_63_]_1-x–y_[Ge_30_Se_70_]_x_Sb_y_ and (**c**) [Ge_30_Se_70_]_1-x–y_[Ge_52_Te_48_]_x_Sb_y_ films. The dots’ size denotes the error bars estimated on the compositions of the films and the dots’ colour corresponds to an estimation of their limit temperature of stability defined by means of the temperature-resolved reflectivity measurements (see “Methods” and the Supplementary Information). Compounds' compositions indicated in square bracket correspond to those of the sputtering targets which may differ from those obtained for the deposited films.

All compositions of the studied chalcogenide thin films are reported on the ternary diagrams of Fig. 1 (see also Sect. 1 of the Supplementary Information). As shown in Fig. 1 the wide composition range of amorphous chalcogenide thin films that can be obtained exhibits a minimal thermal stability of 250 °C and up to higher than 400 °C depending on film composition (see “Methods” and Sect. 2 of the Supplementary Information).

### Amorphous structure of chalcogenide thin films by FTIR and Raman spectroscopies

A summary of the bonds nature and main structural motifs detected in the amorphous structure of chalcogenide thin films deposited by co-sputtering is listed hereafter. All the details are given in Sect. 3 of the Supplementary Information. Table 1 summarizes the main structural motifs for each type of chalcogenide thin film compounds of the present study deduced from analysis of the Raman and FTIR vibration modes as well as the references from literature supporting our conclusions. As follows, the main conclusions on the amorphous structure drawn from the analysis of the Raman and FTIR spectra of each film is summarized system by system based on the extended discussion of the Supplementary Information.

**Table 1:** List of bonds and their main vibration frequencies in the corresponding structural motifs as detected by Raman and FTIR analysis of chalcogenide thin films.

**System**	**Bond**	**Main structural motifs**	**Raman modes** **(cm**^**−1**^**)**		**References**	**IR modes**	**References**
						**(cm**^**−1**^**)**	
Ge_1-x_Se_x_	Ge–Se	GeSe_4/2_ tetrahedra	115; 140		^68,69^	115; 260; 285; 310	^69^
		CS GeSe_4/2_ tetrahedra	195		^70^	–	–
		ES GeSe_4/2_ tetrahedra	218; 310		^70^	–	–
		ETH Ge_2_Se_6_ units	–		–	220	^69^
	Ge–Ge	ETH Ge_2_Se_6_ units	179; 270		^24,70^	–	–
		Ge tetrahedra in amorphous Ge phase	275		^71^	–	–
	Se–Se	Se–Se bridge between GeSe_4/2_ tetrahedra	265		^14^	–	–
Ge_1-x_S_x_	Ge–S	GeS_4/2_ tetrahedra	115; 150		^72,73,74^	147; 367; 388	^75,76^
		CS GeS_4/2_ tetrahedra	343; 425		^70,72,76^	343	^75^
		ES GeS_4/2_ tetrahedra	370; 437		^70,72,76^	437	^75^
	Ge–S–Ge	GeS_4/2_ tetrahedra	–		–	265	^32^
	Ge–Ge	ETH Ge_2_S_6_ units	250		^30,70^	–	–
	S–S	S rings	220; 475		^73^	–	–
		S chains	485		^32^	–	–
Ge_1-x-y_S_x_Se_y_	Ge–Se	GeS_1_Se_3_ mixed tetrahedra	218		^34,35^	–	–
		GeS_2_Se_2_ mixed tetrahedra	232		^34,35^	–	–
		GeS_3_Se_1_ mixed tetrahedra	265		^35^	–	–
	Ge–S	GeS_1_Se_3_ mixed tetrahedra	392		^35^	–	–
		GeS_2_Se_2_ mixed tetrahedra	383		^35^	–	–
		GeS_3_Se_1_ mixed tetrahedra	367		^35^	–	–
Ge_1-x-y_Sb_x_Se_y_	Sb–Se	SbSe_3/2_ pyramids	190		^14^	180; 200; 250	^32,77^
	Sb–Sb	ETH Sb_2_Se_4_ units	159		^14^	156	^78^
		amorphous Sb phase	140		^79,80^	–	–
	Ge–Sb	amorphous Ge_15_Sb_85_ phase	140		^81^	–	–
Ge_1-x-y_Sb_x_S_y_	Sb–S	SbS_3/2_ pyramids	280; 308		^82^	285; 330	^83,84^
	Sb–Sb	ETH Sb_2_S_4_ units	170		^40^	–	–
	Ge–Sb	S_3_Ge–SbS_2_ units	205		^85^	–	–
Ge_1-x-y_Se_x_Te_y_	Ge–Te	GeTe_4/2_ tetrahedra			–	150; 220	^86^
	Ge–Te	Ge–GeTe_3_ tetrahedra or GeTe defective octahedral motifs	120		^87^	–	–
	Se–Te	Se–Te–Se bridge between GeSe_4/2_ tetrahedra	200		^22,88^	–	–
	Te–Te	Te chains	150		^89^	–	–
		amorphous Te phase	157		^90,91^	–	–
Ge_1-x–y-z_ Sb_x_Se_y_Te_z_	Sb–Te	SbTe_3/2_ pyramids	145		^92^	–	–
Si substrate	Si–Si	c–Si modes	300; 520		^93^	–	–

The frequencies of the Raman and FTIR modes correspond to position of experimental peaks reported for stoichiometric glasses in the literature. The analysis of these peaks is extensively discussed in Sect. 3 of the Supplementary Information. CS, ES and ETH denotes respectively corner-sharing, edge-sharing and ethane-like motifs as commonly labelled in the literature.

### Ge_1-x_Se_x_, Ge_1-x_S_x_ and [Ge_40_S_60_]_1-x_[Ge_26_Se_74_]_x_ thin films

**Figure 2:**
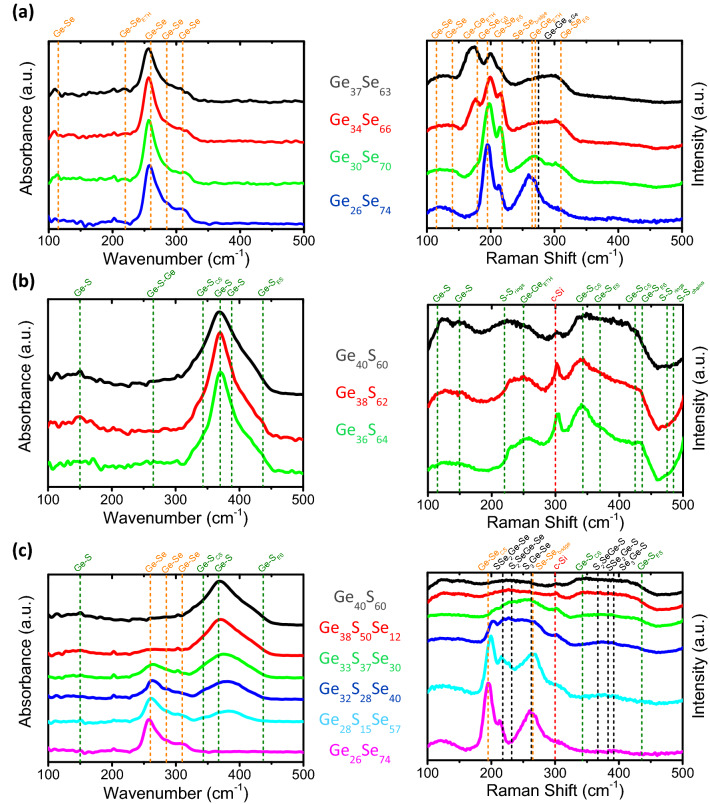
FTIR and Raman spectra of as-deposited (**a**) Ge_1-x_Se_x_ (**b**) Ge_1-x_S_x_ and (**c**) [Ge_40_S_60_]_1-x_[Ge_26_Se_74_]_x_ thin films. The different contribution of vibration modes related to GeSe_2_ and GeS_2_ amorphous phases are indicated by orange and green dash-lines, respectively. The contribution of the c-Si substrate to the Raman signal appearing on some spectra is also indicated by a red dash-line at 300 cm^-1^ as well as a shoulder visible after 450 cm^-1^ that corresponds to a c-Si phonon mode near 520 cm^-1^.

In Fig. 2a are shown the FTIR and Raman spectra acquired on the Ge_1-x_Se_x_, thin films, with x varying in 0.63–0.74 range. Se enrichment in Ge_1-x_Se_x_ films leads to reduction of homopolar inter-tetrahedral Ge–Ge bonds (Ge-Ge_ETH_) in favour of Se–Se ones accompanied by an increase of the number of corner-sharing (CS) compared with edge-sharing (ES) GeSe_4/2_ tetrahedra in excellent agreement with previous works^30^.

The FTIR and Raman spectra of Ge_1-x_S_x_ films are presented in Fig. 2b. In amorphous Ge_1-x_S_x_ films, vibrational modes corresponding to GeS_4/2_ tetrahedra are observed along with contributions attributed to Ge–Ge and S–S homopolar bonds. The decrease of disorder in the amorphous Ge_1-x_S_x_ films evidenced by a hardening of the Raman modes is observed when going from Ge_40_S_60_ toward Ge_36_S_64_ composition as the amorphous network evolves toward composition being more and more close to that of the stoichiometric GeS_2_ glass. The intensity of the visible contribution of the crystalline Si substrate (c-Si) depends on the thickness and transparency of the chalcogenide films at 532 nm.

In Fig. 2c, the FTIR and Raman spectra of the [Ge_40_S_60_]_1-x_[Ge_26_Se_74_]_x_ thin films obtained by co-sputtering of Ge_40_S_60_ and Ge_26_Se_74_ targets exhibit the main modes related to Ge–S and Ge–Se bonds with broadening and a slight frequency shift of their intensity maxima compared with those detected in pure amorphous Ge_40_S_60_ and Ge_26_Se_74_ compounds^31^. The relative intensities of these two main contributions depend on the Ge_40_S_60_/Ge_26_Se_74_ concentration ratio introduced in the film during co-sputtering. Besides, random incorporation of sulphur and selenium in mixed GeS_4-m_Se_m_ tetrahedra (with m = 1, 2 and 3) is observed as supported by previous experimental and simulation studies^32–35^.

### [Ge_30_Se_70_]_1-x_Sb_x_, [Ge_37_S_63_]_1-x_Sb_x_ and [Ge_37_S_63_]_1-x–y_[Ge_30_Se_70_]_x_Sb_y_ thin films

**Figure 3:**
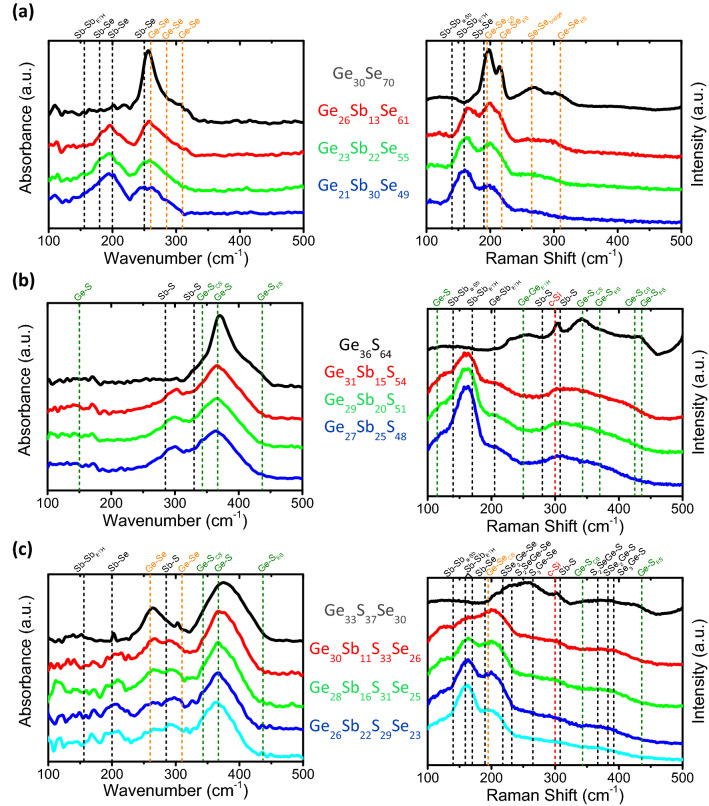
FTIR and Raman spectra of (**a**) [Ge_30_Se_70_]_1-x_Sb_x_ (**b**) [Ge_37_S_63_]_1-x_Sb_x_ and (**c**) [Ge_37_S_63_]_1-x–y_[Ge_30_Se_70_]_x_Sb_y_ thin films. The position of the main vibration modes related to GeSe_2_ and GeS_2_ stoichiometric glasses are indicated by orange and green dash-lines, respectively. The contribution of the c-Si substrate to the Raman signal appearing on some spectra is also indicated by a red dash-line at 300 cm^-1^ as well as a shoulder visible after 450 cm^-1^ that corresponds to a c-Si phonon mode near 520 cm^-1^.

Figure 3a shows the Raman and FTIR spectra acquired on the [Ge_30_Se_70_]_1-x_Sb_x_ films. Addition of Sb in Ge_30_Se_70_ films results in formation of SbSe_3/2_ pyramids, which are reminiscent of the main structural units of amorphous Sb_2_Se_3_, connected to GeSe_4/2_ tetrahedral units by the Se atoms, and a decreasing number of Se–Se homopolar bonds in favour of a more and more prominent amount of homopolar Sb–Sb and wrong Ge–Sb bonds. This observation is of a major importance since homopolar Sb–Sb and wrong Ge–Sb bonds are shown to play a major role on electronic and hence optical properties of chalcogenide glasses^3,36,37^. Apart the non-negligible amount of Sb–Sb and Ge–Sb bonds present in our films which are chalcogen-deficient compared with the GeSe_2_ and Sb_2_Se_3_ stoichiometric compositions, a similar trend has been previously observed in [GeSe_2_]_1-x_[Sb_2_Se_3_]_x_ bulk glasses^38^.

The FTIR and Raman spectra of the [Ge_37_S_63_]_1-x_Sb_x_ thin films are shown in Fig. 3b. S-deficient [Ge_37_S_63_]_1-x_Sb_x_ films are mainly composed of GeS_4/2_ tetrahedral units and, upon addition of pure Sb, Sb–Sb and Sb–Ge bonds are formed with appearance of a small amount of SbS_3/2_ pyramids. Therefore, in other words the amorphous network of our S-poor glasses can be depicted as a mix of GeSb_4-m_S_m_ tetrahedral motifs (with m = {0, 1, 2, 3, 4}) and few SbGe_3-m-n_Sb_m_S_n_ pyramidal units (with n + m = {0, 1, 2, 3}) in favour of homopolar Sb–Sb and wrong Sb–Ge bonds^39–42^.

In Fig. 3c, the FTIR and Raman spectra of the [Ge_37_S_63_]_1-x_[Ge_30_Se_70_]_x_ thin films upon Sb incorporation show vanishing of the Raman modes of mixed GeS_4-m_Se_m_ tetrahedral units at least in favour of an increase number of GeSe_4/2_ tetrahedra. This indicates differences between Ge and Sb atoms in chemical bonding affinity with S and Se chalcogen elements. This has a different impact on the amorphous structure depending on the S/Se ratio. Increasing Sb concentration in [Ge_37_S_63_]_1-x–y_[Ge_30_Se_70_]_x_Sb_y_ films leads to preferential formation of Ge–Se and Sb–S bonds by detriment to Ge–S and Sb–Se ones in films exhibiting a lack of chalcogen element compared with the stoichiometric compositions in agreement with a previous study^41^. Raman and in a less manner FTIR spectra also evidences the presence of a significant amount of Ge–Ge, Sb–Sb and Ge–Sb bonds in our sputtered [Ge_37_S_63_]_1-x–y_[Ge_30_Se_70_]_x_Sb_y_ films.

### [Ge_30_Se_70_]_1-x_[Ge_52_Te_48_]_x_, [Ge_30_Se_70_]_1-x–y_[Ge_52_Te_48_]_x_Sb_y_ and [Ge_1-2x_Se_x_Te_x_]_1-y_Sb_y_ thin films

**Figure 4:**
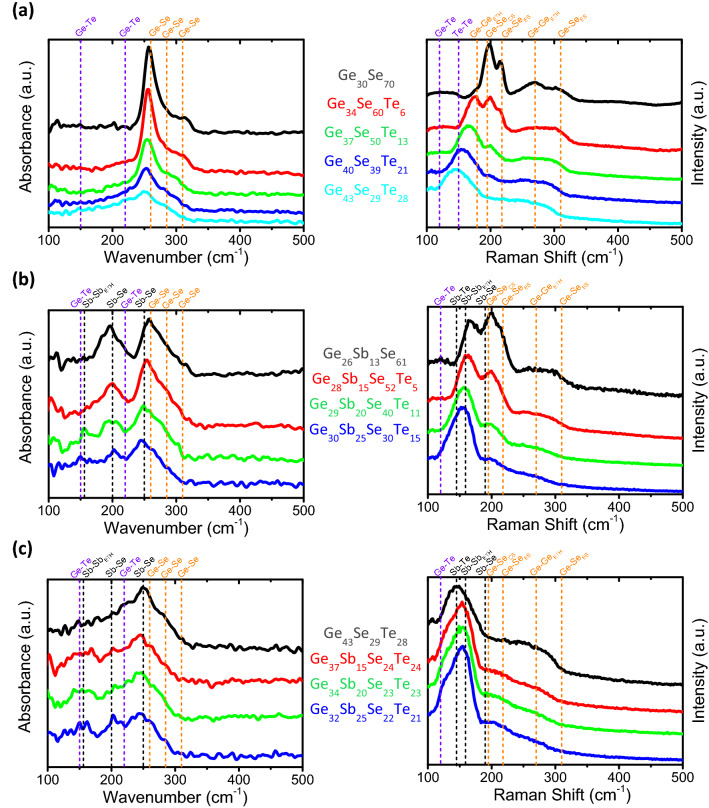
FTIR and Raman spectra of (**a**) [Ge_30_Se_70_]_1-x_[Ge_52_Te_48_]_x_, (**b**) [Ge_30_Se_70_]_1-x–y_[Ge_52_Te_48_]_x_Sb_y_ and (**c**) [Ge_1-2x_Se_x_Te_x_]_1-y_Sb_y_ thin films. The position of the main modes related to amorphous GeTe or GeSe_2_ compounds as well as those related to Sb atoms are indicated by orange, purple and black dash-lines, respectively.

Figure 4a shows the FTIR and Raman spectra acquired on the [Ge_30_Se_70_]_1-x_[Ge_52_Te_48_]_x_ thin films obtained by co-sputtering of Ge_30_Se_70_ and Ge_52_Te_48_ targets. Ge-GeTe_3_ tetrahedra and GeTe defective octahedral motifs present in a-GeTe phase^43,44^ are expected also in Ge_52_Te_48_-rich [Ge_30_Se_70_]_1-x_[Ge_52_Te_48_]_x_ films. The structure of our [Ge_30_Se_70_]_1-x_[Ge_52_Te_48_]_x_ thin films obtained by co-sputtering can be depicted as GeSe_4-n_Te_n_ tetrahedral motifs with n = {0, 1, 2, 3, 4} forming a disordered network connected, for small x values, by means of the chalcogen elements and, for higher x values, coexisting with a non-negligible amount of Ge–Ge homopolar bonds and possibly some Te–Te bonds as well as Se–Te bonds as proposed in a previous work^22^.

In Fig. 4b,c are shown the FTIR and Raman spectra of [Ge_30_Se_70_]_1-x–y_[Ge_52_Te_48_]_x_Sb_y_ and [Ge_1-2x_Se_x_Te_x_]_1-y_Sb_y_ thin films obtained by co-sputtering of Ge_30_Se_70_, Ge_52_Te_48_ and Sb targets. The comparison between both figure tends to indicate that in our amorphous [Ge_30_Se_70_]_1-x_[Ge_52_Te_48_]_x_ films, which are getting more and more Ge-rich as x value is increased, Ge–Ge homopolars are found and Ge–Se(Te) bonds represent the vast majority of bonds involving the chalcogen elements. Upon introduction of Sb in the [Ge_30_Se_70_]_1-x_[Ge_52_Te_48_]_x_ films, whereas the contribution of Sb–Se bonds to the Raman spectra is weak their presence in IR absorption is more visible in FTIR spectra probably thanks to the high IR cross section of such bonds compared with others. Sb doping of the Ge_43_Se_29_Te_28_ compound lead to formation of Sb-Sb homopolar bonds and probably Ge–Sb wrong bonds as already discussed above for Sb-doped Ge_30_Se_70_ films. As a result, [Ge_30_Se_70_]_1-x–y_[Ge_52_Te_48_]_x_Sb_y_ films obtained by co-sputtering appear to be a highly disordered system. It can be depicted as a mix of Ge–GeTe_3_ and GeSb_4-m-n_Se_m_Te_n_ tetrahedra (with m + n = {0, 1, 2, 3, 4}) as well as SbGe_3-m-n_Se_m_Te_n_ motifs (with m + n = {0, 1, 2, 3}). The relative fraction between these motifs varies as a function of the Ge/Se/Te atomic ratio of the films.

To conclude, the effect of Sb incorporation in the amorphous structure of [Ge_37_S_63_]_1-x_[Ge_30_Se_70_]_x_ and [Ge_30_Se_70_]_1-x_[Ge_52_Te_48_]_x_ thin films is different depending on the nature of the chalcogen elements present in the film. A clear difference is observed between films containing S/Se compared with Se/Te as chalcogen elements. In particular, this is evidenced by formation of GeSb_4-m-n_Se_m_Te_n_ tetrahedra (with m + n = {0, 1, 2, 3, 4}) as well as SbGe_3-m-n_Se_m_Te_n_ motifs (with m + n = {0, 1, 2, 3}) in Sb-doped [Ge_30_Se_70_]_1-x_[Ge_52_Te_48_]_x_ thin films whereas no such mixed Ge tetrahedra nor mixed Sb pyramids can be observed in [Ge_37_S_63_]_1-x_[Ge_30_Se_70_]_x_ compounds doped with Sb. Besides, in both case Sb addition [Ge_37_S_63_]_1-x_[Ge_30_Se_70_]_x_ and [Ge_30_Se_70_]_1-x_[Ge_52_Te_48_]_x_ films does not lead to a random and homogeneous distribution of the chalcogen atoms in Ge-centered tetrahedra as observed in Ge-based chalcogenide films. From all the above FTIR and Raman study, one can conclude that the local order and the structure of the (co-)sputtered chalcogenide thin films are shown to largely vary with in particular a very different amount of homopolar bonds and the latter is shown to depend on thin films’ atomic composition. Since the properties of materials being intimely linked to their structure, probing the link between structure and optical properties is an invaluable clue in order to propose design rules aiming at fabricating chalcogenide compounds thin films with optimized properties for the applications in photonics. In the following, the linear and nonlinear optical constants of the films, such as the real and imaginary part of refractive index as well as Kerr nonlinear refractive index, are studied.

### Optical properties of the chalcogenide thin films

#### Linear optical constants

**Figure 5:**
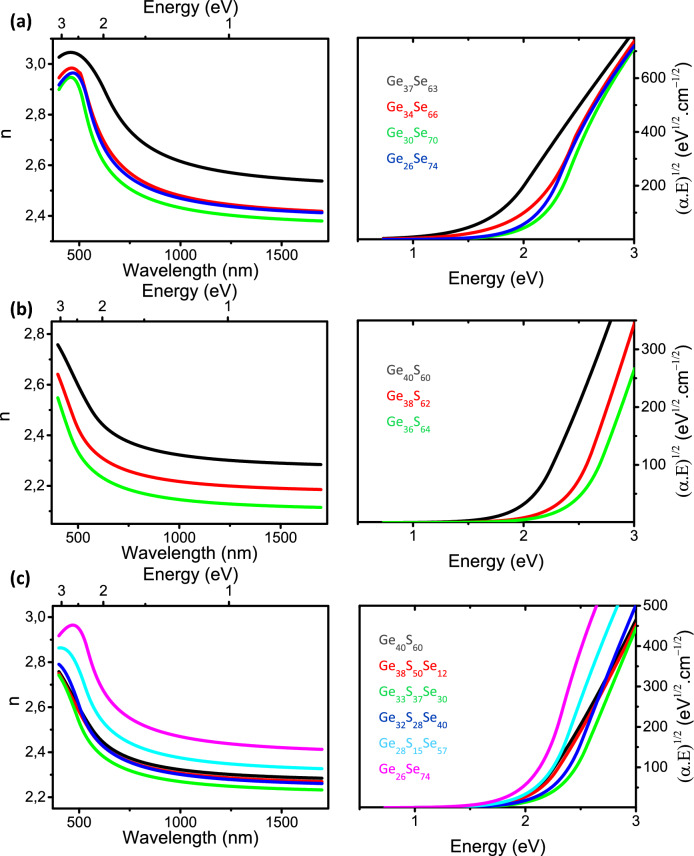
Refractive index n (left panels) and Tauc’s plots (right panels) of (**a**) Ge_1-x_Se_x_ (**b**) Ge_1-x_S_x_ and (**c**) [Ge_40_S_60_]_1-x_[Ge_26_Se_74_]_x_ thin films deposited by (co-)sputtering.

**Figure 6:**
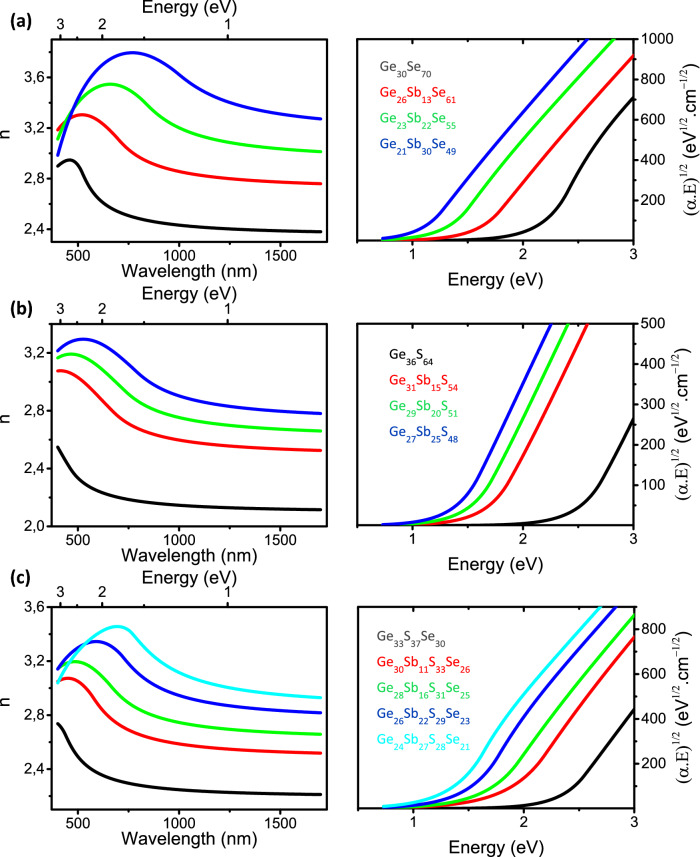
Refractive index n and Tauc’s plots of (**a**) [Ge_30_Se_70_]_1-x_Sb_x_ (**b**) [Ge_37_S_63_]_1-x_Sb_x_ and (**c**) [Ge_37_S_63_]_1-x–y_[Ge_30_Se_70_]_x_Sb_y_ thin films deposited by (co-)sputtering.

**Figure 7:**
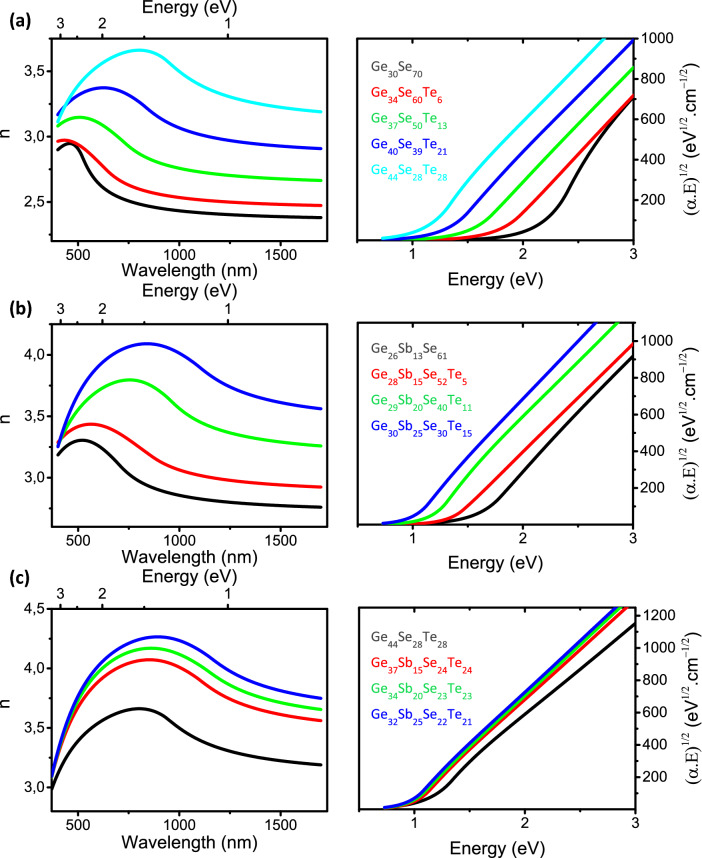
Refractive index n and Tauc’s plots of (**a**) [Ge_30_Se_70_]_1-x_[Ge_52_Te_48_]_x_ (**b**) [Ge_30_Se_70_]_1-x–y_[Ge_52_Te_48_]_x_Sb_y_ and (**c**) [Ge_1-2x_Se_x_Te_x_]_1-y_Sb_y_ thin films deposited by (co-)sputtering.

The optical constants (refractive index n and extinction coefficient k) of the films were deduced from the spectroscopic ellipsometry measurements from visible to near-IR (NIR) range (see “Methods”). The refractive indices n and Tauc’s plots of (α.E)^1/2^ vs energy (eV) obtained from the extinction coefficient k (see “Methods”) are plotted in Figs. 5, 6, 7 for each composition of the chalcogenide films. First, Figs. 5, 6, 7 call for a general comment. For wavelength range located above inter band absorption range, which corresponds to energies lower than the bandgap energy (see the Tauc’s plots of Figs. 5, 6, 7), the refractive indices tend progressively to a kind of plateau. The latter gives therefore an estimation of the refractive indices in the MIR range up to multi-phonons absorption appearing at significantly higher wavelengths than NIR^45,46^. Therefore, the study of optical properties in the visible-NIR range is the best compromise in order to get an estimation of refractive indices from visible to MIR range as well as giving an estimation of the optical band gap energy from absorption measurements or ellipsometry data fitting models (see “Methods” and Sect. 4 of the Supplementary Information).

### Ge_1-x_Se_x_, Ge_1-x_S_x_ and [Ge_40_S_60_]_1-x_[Ge_26_Se_74_]_x_ thin films

In Fig. 5a, the refractive index of the sputtered Ge_1-x_Se_x_ (0.63 < x < 0.74) thin films in the NIR range (above 850 nm) does not evolve linearly with composition but reaches a maximum for x = 0.63 as well as a minimum for x = 0.7. This effect can be related to the amorphous structure of Ge_37_Se_63_ film which exhibits the highest amount of Ge–Ge homopolar bonds in ethane-like structures among all Ge_1-x_Se_x_ (0.63 < x < 0.74) films (see the Raman of Fig. 2a). Besides, ab initio molecular dynamics simulations reported that the presence of numerous distorted Se–Ge–Se angles in Ge_1-x_Se_x_ (0.60 < x < 0.66) permits to reduce the stress induced by the increase of the Ge content and thus the mean atomic coordination number or network connectivity^47^. By opposite, the Ge_30_Se_70_ film which has the lowest refractive index also presents the lowest amount of Ge–Ge and Se–Se homopolars and the narrowest and most well-defined modes corresponding to CS and ES GeSe_4/2_ tetrahedra (195 and 212 cm^−1^) as shown in Fig. 2a. This trend on the refractive index is in good agreement with previous literature for bulk glasses in which compositions with the highest Ge–Se/(Ge–Ge + Se−Se) bonding ratio exhibited the lowest refractive indices^26^. The absorption of Ge_30_Se_70_ film evidences its highest band gap value among all Ge_1-x_Se_x_ (0.63 < x < 0.74) films as seen on the Tauc’s plots of Fig. 5a. Besides, the Ge_37_Se_63_ composition with the highest refractive index has the lowest band gap energy. The decreasing number of Ge–Ge bonds may be at origin of the strong increase of absorption between Ge_34_Se_66_ and Ge_37_Se_63_ films.

Ge_1-x_S_x_ thin films experience a significant increase of their refractive index in the NIR as the Ge fraction is slightly increased from x = 0.64 to x = 0.60 (Fig. 5b). This trend is consistent with previous literature studies^48,49^. This could be attributed to an increasing number of distorted S–Ge–S bonds similarly to the previously mentioned presence of numerous distorted Se–Ge–Se angles in Ge_1-x_Se_x_ (0.60 < x < 0.66)^47^ related to the increase of the number of Ge–Ge homopolar bonds (see Fig. 2b) with the decrease of the S content. This leads to a reduction of the band gap energy in agreement with previous literature^49^ due to creation of new electronic states under the conduction band explaining the progressive shift of absorption toward lower energy (Fig. 5b)^50^. The absorption and refractive index change monotonously as x increases in our Ge_1-x_S_x_ films (Fig. 5b).

The refractive index of the [Ge_40_S_60_]_1-x_[Ge_26_Se_74_]_x_ films also exhibits a minimum for the Ge_33_S_37_Se_30_ composition (Fig. 5c). This is not surprising since, similarly to Ge_1-x_Se_x_ and Ge_1-x_S_x_ films, the amount of Ge–Ge homopolar bonds is expected to be minimum for this near-stoichiometric composition with a Ge/(S + Se) atomic ratio close to 1/2 (Fig. 2c). As a result, the general trends on refractive indices of studied Ge-based chalcogenide films are driven by the ratio between heteropolar and homopolar bonds.

Among the [Ge_40_S_60_]_1-x_[Ge_26_Se_74_]_x_ thin films, the band gap energy reaches a maximum for the Ge_33_S_37_Se_30_ composition as evidenced by the absorption curves in Fig. 5c. This results from the composition of Ge_33_S_37_Se_30_ film which is close to the GeCh_2_ stoichiometric compound (with Ch referring to S or Se chalcogen element) expected to exhibit almost no homopolar bonds. Note that for a same Ge concentration, replacing S by Se atoms results in a progressive decrease of band gap energy^31^.

### [Ge_30_Se_70_]_1-x_Sb_x_, [Ge_37_S_63_]_1-x_Sb_x_ and [Ge_37_S_63_]_1-x–y_[Ge_30_Se_70_]_x_Sb_y_ thin films

In Fig. 6, replacing tetravalent Ge atoms, as observed in structural motifs of stoichiometric GeSe_2_ glass, by trivalent Sb atoms, as found in amorphous stoichiometric Sb_2_Se_3_ compound, within the GeS_x_Se_1-x_ films increases the non-chalcogen/chalcogen element ratio required to keep the stoichiometric composition. This has been clearly evidenced for composition tie-lines crossing the stoichiometric GeSe_2_/Sb_2_Se_3_ pseudo-binary tie-line as Ge_40-x_Sb_x_Se_60_^51^, Ge_x_Sb_10_Se_90-x_^52,53^ Ge_x_Sb_15_Se_85-x_ or Ge_x_Sb_20_Se_80-x_^53^ glasses. These compositions are again those with the smallest amount of homopolar bonds and corresponding to a limit of topological phase transition as reported in Ge_40-x_Sb_x_Se_60_ glasses^54,55^. Besides, in bulk glasses as well as in thin films, the decrease of the GeSe_2_/Sb_2_Se_3_ ratio was shown to result in an increase of the refractive index and a decrease of the band gap energy since electronic polarizability of Sb–Se bonds is much higher than that of Ge-Se bonds^24,56,57^.

In Fig. 6 are shown the change of optical constants upon Sb addition by means of co-sputtering in the Ge_30_Se_70_, Ge_33_S_67_ and [Ge_33_S_67_]_1-x_[Ge_30_Se_70_]_x_ thin films in order to increase films' refractive indices by drifting away from compositions located on the GeCh_2_/Sb_2_Ch_3_ pseudo-binary tie-line. In Figs. 6a–c the incorporation of Sb in Ge_30_Se_70_, Ge_33_S_67_ and [Ge_33_S_67_]_1-x_[Ge_30_Se_70_]_x_ films significantly increases films’ refractive index and at the same time reducing the optical band gap energy of the material. This effect is attributed to the higher electronic polarizability of Sb atoms in particular when forming Sb–Ch and Sb–Sb bonds. These highly polarizable bonds are revealed to appear in an increasing level as the incorporated Sb amount is increased in films (see amorphous structure analysis detailed in the Amorphous Structure section and in the Supplementary Information). One can also note that among all these compositions, some films exhibit compositions very close to those of well-studied commercial glasses such as for instance AMTIR-3 glass (also commercially called IG5 or IRG-5 or BD-2 or OPTIR-3) of Ge_28_Sb_12_Se_60_ composition. Thus, this well-known glass is close to the Ge_26_Sb_13_Se_61_ film of the present study and can be used as a point of comparison. In Fig. 6a, the optical constant values of our [Ge_30_Se_70_]_1-x_Sb_x_ films are in good agreement with the literature^14,52,53^.

### [Ge_30_Se_70_]_1-x_[Ge_52_Te_48_]_x_, [Ge_30_Se_70_]_1-x–y_[Ge_52_Te_48_]_x_Sb_y_ and [Ge_1-2x_Se_x_Te_x_]_1-y_Sb_y_ thin films

Figure 7a shows that, similarly to Sb, introduction of Ge_52_Te_48_ in [Ge_30_Se_70_]_1-x_[Ge_52_Te_48_]_x_ films results in a huge increase of refractive index of the Ge_30_Se_70_ glass. As in the Sb doping, this effect could be attributed to the higher electronic polarizability of atoms of Ge–Te motifs compared with those in Ge-Se ones, and that form in a growing concentration in [Ge_30_Se_70_]_1-x_[Ge_52_Te_48_]_x_ films upon increasing the Ge_52_Te_48_ concentration (see Fig. 4a). Moreover, the refractive index of [Ge_30_Se_70_]_1-x_[Ge_52_Te_48_]_x_ films can be further increased by Sb incorporation as shown in Fig. 7b,c. Such an increase is near directly correlated to the concentration of Te and Sb atoms in the [Ge_30_Se_70_]_1-x–y_[Ge_52_Te_48_]_x_Sb_y_ and [Ge_1-2x_Se_x_Te_x_]_1-y_Sb_y_ films with a more and more significant effect when highly polarizable environments such as Sb–Te, Sb-Sb bonds or in a less extent Ge–Te bonds form in the amorphous material^58^.

In Fig. 7a, the increase of absorption of [Ge_30_Se_70_]_1-x_[Ge_52_Te_48_]_x_ films upon increasing x can be related to the smallest bandgap of a-GeTe compared to that of Se-based compound^59^. The absorption progressively decreases as the band gap of the film increases upon moving toward the Ge_30_Se_70_ composition. By opposite, in Fig. 7b,c for [Ge_30_Se_70_]_1-x–y_[Ge_52_Te_48_]_x_Sb_y_ films, a more complex trend is observed. First, in Fig. 7b an increase of the (Sb + Te)/Se ratio in [Ge_30_Se_70_]_1-x–y_[Ge_52_Te_48_]_x_Sb_y_ films lead to a shift of the absorption curve toward lower energy due to a decrease of band gap energy. More surprising, in Fig. 7c the absorption curves and optical band gap of [Ge_1-2x_Se_x_Te_x_]_1-y_Sb_y_ films remain almost constant for the three compounds containing Sb concentration ranging from 15 to 30 at. %. Increasing the concentration of homopolar bonds, mainly Sb-Sb ones, and Te-related bonds due to an increase of the (Sb + Te)/Se ratio in [Ge_30_Se_70_]_1-x–y_[Ge_52_Te_48_]_x_Sb_y_ films result in an increase of band tails as well as creation of electronic states in the bandgap, and hence to a reduction of the optical bandgap energy.

However, in Fig. 7c upon incorporation of Sb in the ternary Ge_1-2x_Se_x_Te_x_ compound, the refractive index of the films significantly increase while the decrease in bandgap energy is negligible. The explanation is related to the changes in the amorphous structure. First, the increasing concentration of Sb leads to an increase of the material polarizability and thus that of the refractive index. However, at the same time the amount of Te–Ge heteropolar bonds decreases leading to an increase of band gap energy of the material hence counterbalancing the effect of Sb. Therefore in [Ge_1-2x_Se_x_Te_x_]_1-y_Sb_y_ films, the incorporation of highly polarizable Sb bonds leads to an increase of the refractive index but does not significantly affect the optical band gap energy value. Indeed, the replacement of Ge–Ge homopolars by Sb–Sb bonds upon Sb introduction is expected to impact mostly the density and nature of localized electronic defect states in the material band gap but with no or limited effect on the bandgap energy.

To summarize, the observed trend of refractive indices as a function of chalcogenide thin films’ compositions can be reasonably correlated to changes in films’ amorphous structure as extensively described in the FTIR/Raman experiments of the Amorphous Structure section and in Sect. 3 of the Supplementary Information. The electronic polarizability of local chemical environments and bonding configurations in the amorphous is shown to play key role aiming at controlling the refractive indices of the films. In particular, introduction of highly polarizable bonds such as Sb–Sb homopolars, Sb–Ch (with Ch = Te or Se) or Te-X (with X = Sb or Ge) and in a less extent Ge–Ge bonds leads to a significant increase of the refractive index concomitant to a decrease of the optical bandgap energy. The optical band gap energy is also an outmost parameter that should be taken into account for MIR and nonlinear photonic applications regarding for instance transparency window, two-photon absorption (TPA) losses and so on. The band gap energy values of the chalcogenide thin films are listed in Table 2. The obtained band gap energies and refractive index values are found to follow the well-known Moss rule^60^, which relates the refractive index (or optical dielectric constant) to the optical band gap energy of semiconductors^61^.

**Table 2:**
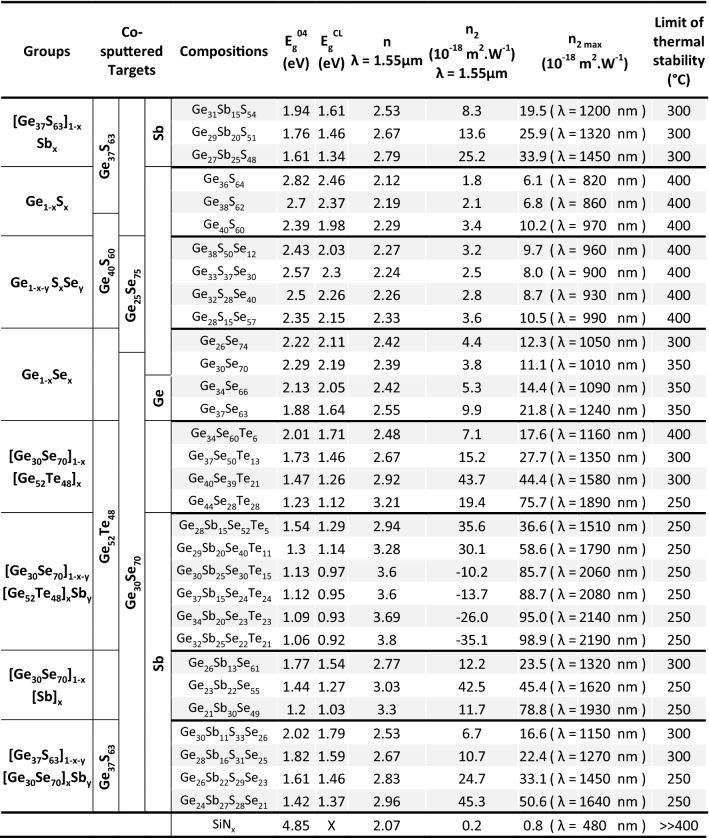
Summary of chalcogenide thin films obtained by (co-)sputtering deposition: composition, nature of the sputtering targets used for (co-)sputtering deposition, refractive index at 1.55 µm, band gap energy E_g_^04^ and E_g_^CL^, n_2_ Kerr nonlinear refractive index calculated either by means of the Sheik-Bahae model at 1.55 µm (n_2_) and maximal n_2_ values (n_2 max_) at energy near 0,534 × E_g_^opt^ eV (the corresponding wavelength value is also indicated into brackets) and a first evaluation of the limit temperature for material’s stability (see text for details as well as “Methods” and Sect. 2 of the Supplementary Information).

In order to evaluate the potential interest for applications of such chalcogenide thin films in the emerging field of on-chip nonlinear photonics, determining the Kerr refractive indices, which quantify the nonlinear frequency conversion efficiencies, can give a first interesting insight. For that purpose, in the following the Kerr refractive indices of the films are estimated by means of their linear optical constants as well as using estimated values of the band gap energy.

#### Kerr nonlinear refractive indices n_2_

The n_2_ Kerr nonlinear refractive indices were evaluated mainly using the Sheik–Bahae model^29^. We considered this model as enough accurate in order to get qualitative trends on n_2_ values (see Sect. 5 of the Supplementary Information). It has to be emphasized that a systematic error of about 20% exists in the experimental determination of non-linear optical coefficients.

The Kerr refractive index is strongly correlated with the linear refractive index and the optical band gap energy value. Using the Sheik–Bahae model, the maximum of the third order nonlinear parameter is found for an energy close to 0,534 × E_g_^opt^. Therefore, the values of n_2_ can vary considerably depending on the wavelength and must be taken into consideration depending on the value of the wavelength that will be used in the application. However, previous experimental work reported a maximum of Kerr index for photon energy values higher than that corresponding to 0,534 × E_g_^opt^ ^53^. Table 2 shows the maximum values of the n_2_ Kerr refractive indices for all the films studied in this work and their corresponding wavelengths, as well as the values obtained at 1,550 nm, the standard wavelength for telecommunications. In order to validate the Sheik–Bahae model, this method was also applied to samples of silica and silicon nitride thin film. The n_2_ values at 1,550 nm for these two reference materials have been calculated to be 3 × 10^–20^ and 2 × 10^–19^ m^2^/W, respectively. These values are in excellent agreement with those experimentally measured in the literature^60^. Therefore, one can conclude that in our (co-)sputtered amorphous chalcogenide thin films, the Kerr indices are of two to three orders of magnitude higher than those obtained for silica or silicon nitride materials. These values are also in excellent agreement with previous experimental results reported for similar Ge_1-x-y_Sb_x_Se_y_, Ge_1-x-y_Sb_x_S_y_ compounds^38,63–65^ as well as those deduced using the Sheik–Bahae model for Ge_1-x_S_x_ and Ge_1-x-y_Sb_x_Se_y_ glasses^49,66^.

For some of the chalcogenide thin films, the Sheik–Bahae model gives negative Kerr indices at 1,550 nm. In previous works, only positive experimental n_2_ values were observed in the transparency window of chalcogenide glasses^61,67^. Nevertheless, negative Kerr indices values were reported in Ge_1-x-y_Sb_x_Se_y_ glasses at 800 nm^66^ where absorption starts to be non-negligible. Therefore, we can emphasize that the negative Kerr indices values at 1.55 µm (~ 0.8 eV) are found for co-sputtered films for which the optical band gap energy is below 1.6 eV and as a result when TPA becomes significant.

In literature, n_2_ values have been related to the amorphous structure. In particular, a clear correlation was found between n_2_ values and the concentration of highly polarizable heteropolar bonds^38^. Herein, one observe a clear correlation between material polarizability, which is proportional to the linear refractive index, and the nonlinearities evidenced by the n_2_ values calculated by using the Sheik-Bahae model. Thus, one can relate the enhancement of electronic polarizability and the resulting increase of n_2_ values to presence of peculiar local atomic motifs and bonding configurations found in the amorphous structure of films. For instance, the amount of homopolar bonds play a key role in the increase of linear and nonlinear refractive indices as described above.

Finally, in Table 2 are also reported for each films the limit of temperature after which a degradation of the material could be observed by monitoring optical reflectivity at 670 nm upon annealing (see “Methods” and Sect. 2 of the Supplementary Information). This is also very instructive since temperature limit in between 250 and 400 °C were obtained. This emphasizes that a compromise between optical properties and thermal stability has to be found for selection of a particular composition depending on final application as well as taking into account the thermal budget required for integration process flow in devices. Indeed, an annealing after deposition is also expected to significantly affect the optical properties of these Ge-based amorphous chalcogenide thin films due to the structural relaxation. Thus, aging of these metastable amorphous materials will have to be studied in the future aiming at ensuring durability of MIR components that would integrate some of these compounds.

## Conclusion

To conclude, industrial co-sputtering deposition method is a powerful tool in order to fastly study a wide compositions range of amorphous chalcogenide thin films aiming at ultimately achieving highly nonlinear on-chip MIR components. By means of a systematic study of the amorphous structure correlated with the trend on optical properties (linear optical constants, optical band gap and Kerr nonlinear refractive index) of the as-deposited chalcogenide thin films one can get invaluable clues in order to optimize the materials optical properties towards future applications. The materials’ polarizability and thus linear and nonlinear refractive indices increase significantly when moving from light to heavier chalcogen element such as S to Se and toward Te-based chalcogenide compounds but accompanied with a decrease of the thermal stability. The ratio of homopolar/wrong on heteropolar bonds in the amorphous chalcogenide is shown to play a main role on the electronic and thus optical properties of the films. For instance, the introduction of Sb atoms deeply modifies the amorphous structure as well as introducing Sb–Sb homopolar bonds. As a result, the electronic polarizability of the glass is significantly enhanced as evidenced by the significant increase of the material refractive indices. However, this also leads to a detrimental decrease of the material’s thermal stability and bandgap. Moreover, we show that the outstanding and state-of-the-art Kerr refractive indices in the MIR range are found for chalcogenide thin films deposited by means of industrial sputtering technique enabling fast transfer to applications. Finally, we demonstrate that a good trade-off between high nonlinearity, good thermal stability and optimized working wavelength in the IR can be found opening wide range of opportunities for future on-chip photonic applications fully compatible with CMOS large-scale integration technologies.

## Electronic supplementary material

Supplementary information
