# Supplementary material for: Ge–Sb–S–Se–Te amorphous chalcogenide thin films towards on-chip nonlinear photonic devices
Source: Sci Rep. 2020 Jul 17;10:11894. doi: 10.1038/s41598-020-67377-9 (PMC7367863; doi:10.1038/s41598-020-67377-9)
Supplement: Supplementary file 1 — Supplementary information [file 41598_2020_67377_MOESM1_ESM.pdf]

Supplementary Information

Ge-Sb-S-Se-Te amorphous chalcogenide thin films towards on-chip nonlinear photonic devices

J.-B. Dory[1], C. Castro-Chavarria[1], A. Verdy[1], J.-B. Jager[2], M. Bernard[1], C. Sabbione[1], M. Tessaire[1], J.-M. Fédéli[1], A. Coillet[3], B. Cluzel[3] and P. Noé[1,*]

[1] Université Grenoble Alpes, CEA, LETI, MINATEC Campus, 17 avenue des Martyrs, F 38000 Grenoble, France.

[2] Université Grenoble Alpes, CEA, INAC, MINATEC Campus, 17 avenue des Martyrs, F 38000 Grenoble, France.

[3] ICB, UMR CNRS 6303, Université de Bourgogne Franche Comté, 9, avenue Alain-Savary, BP 47870, 21078 Dijon cedex, France.

*Contact: pierre.noe@cea.fr

1. **Working powers of sputtering targets and composition ranges of studied thin films**
2. **Analysis of thermal stability limit of the (co)- sputtered amorphous chalcogenide thin films**
3. **Analysis of amorphous structure of chalcogenide thin films by FTIR and Raman spectroscopies**
   - ***3.1. $Ge_{1-x}Se_x$, $Ge_{1-x}S_x$ and $[Ge_{40}S_{60}]_{1-x}[Ge_{26}Se_{74}]_x$ thin films***
   - ***3.2. $[Ge_{30}Se_{70}]_{1-x}Sb_x$, $[Ge_{37}S_{63}]_{1-x}Sb_x$ and $[Ge_{37}S_{63}]_{1-x-y}[Ge_{30}Se_{70}]_xSb_y$ thin films***
   - ***3.3. $[Ge_{30}Se_{70}]_{1-x}[Ge_{52}Te_{48}]_x$, $[Ge_{30}Se_{70}]_{1-x-y}[Ge_{52}Te_{48}]_xSb_y$ and $[Ge_{1-2x}Se_xTe_x]_{1-y}Sb_y$ thin films***
4. **Modelling of the spectroscopic ellipsometry data and the Cody-Lorentz model**
5. **n_2 nonlinear refractive indices determination by means of the Sheik-Bahae model**

1. Working powers of sputtering targets and composition ranges of studied thin films

Compositions	Targets	Powers (W)		
		DC/RF	Min.	Max.
$Ge_{1-x-y-z}S_xSe_yTe_z$	Te	DC	11	50
	$Ge_{40}S_{60}$	RF	25	150
	$Ge_{25}Se_{75}$	RF	10	100
$Ge_{1-x-y}Sb_xSe_y$	Sb	DC	15	50
	Ge	DC	16	33
	$Ge_{30}Se_{70}$	RF	220	
$Ge_{1-x-y-z}Sb_xS_ySe_z$	Sb	DC	15	
	$Ge_{33}S_{67}$	RF	60	178
	$Ge_{30}Se_{70}$	RF	60	200
$Ge_{1-x-y-z}Sb_xSe_yTe_z$	Sb	DC	15	
	$Ge_{50}Te_{50}$	RF	30	170
	$Ge_{30}Se_{70}$	RF	66	220

Table S1. Range of sputtering powers applied to chalcogenide pure or composite targets used for deposition of chalcogenide thin films.

Two families and 8 groups of compositions are studied:

- A first class of germanium-based amorphous films divided into four groups depending on the nature of the chalcogen elements:
  - $Ge_{1-x}Se_x$, with x varying in 0.63-0.74 range ;
  - $Ge_{1-x}S_x$, with x varying in 0.6-0.64 range ;
  - $[Ge_{40}S_{60}]_{1-x}[Ge_{26}Se_{74}]_x$ with x varying in 0.17-0.75 ranges;
  - $[Ge_{30}Se_{70}]_{1-x}[Ge_{52}Te_{48}]_x$ with x varying in 0-0.58 range.
- And a second series of chalcogenide films divided into four groups in order to study the impact of antimony incorporation on the above-mentioned germanium-based chalcogenide glasses:
  - $[Ge_{30}Se_{70}]_{1-x}[Sb]_x$ with x varying in 0-0.3 range ;
  - $[Ge_{37}S_{63}]_{1-x}[Sb]_x$ with x varying in 0-0.25 range ;
  - $[Ge_{37}S_{63}]_{1-x-y}[Ge_{30}Se_{70}]_x[Sb]_y$ with x and y varying in 0.3-0.37 and 0.11-0.27 ranges, respectively ;
  - $[Ge_{30}Se_{70}]_{1-x-y}[Ge_{52}Te_{48}]_xSb_y$ quaternary with:
    - x = 0.58 and y varying in 0-0.25 range ;
    - x and y varying in 0.1-0.31 and 0.13-0.25 ranges, respectively.

2. Analysis of thermal stability limit of the (co)- sputtered amorphous chalcogenide thin films

The limit of thermal stability of all the sputtered chalcogenide films has been assessed either by monitoring any change of their reflectivity upon annealing as well as controlling any significant and irreversible change of their amorphous structure by means of FTIR and Raman spectroscopies or by simply controlling their aspect by means of optical microscopy. The obtained limit temperature for each film are reported in **Table 1** of the main text. Depending on the thin film samples' composition, this limit corresponds either to the crystallization of the film or to a degradation such as delamination of stacking or a phase separation in the film. For instance, Sb segregation leading to Sb clustering in Sb-rich films could be identified by means of micro-Raman measurement as well as formation of pure Se phase in Se-rich films. Such observations are in good agreement with previous works on similar compounds.[1] The annealing of the films above their crystallization temperature result in a slight hardening of the main vibration modes related to a decrease of disorder in the glass network accompanied with a decrease of refractive indices and increase of band gap energy. These observations are consistent with previous works showing similar trend attributed to amorphous phase relaxation upon thermal ageing towards a lower energy glass.[2,3] The main aim of such thermal stability analysis is to give a first evaluation of the limit thermal budget that the films can experience without irreversible change of their properties during integration processes.

Besides, analysis of optical reflectivity curves of the films upon annealing is also very instructive, however concluding on the underlying physical mechanism is difficult and would need further investigations. Nevertheless, some obvious main trends could be derived from such optical measurements. Depending on films' composition slow reflectivity changes corresponding to glass transition region or abrupt reflectivity increase related to crystallization could be observed. Even if such a technique is not the most appropriate to identify such phase transitions, the reflectivity changes identified as glass transition temperatures are consistent with T_g values of literature for similar compositions. For instance for $Ge_{30}Se_{70}$, $Ge_{37}Se_{63}$ $Ge_{34}Se_{66}$ thin films, a slight increase of reflectivity is observed around 335, 400 and 410°C respectively. These temperatures correspond to their expected glass transition temperatures.[4,5] Moreover, the thermal behaviour of $Ge_{34}Se_{60}Te_{6}$ - $Ge_{37}Se_{50}Te_{13}$ - $Ge_{40}Se_{39}Te_{21}$ films is very close to the one observed in previous work studying similar compositions[5]. The $Ge_{26}Sb_{13}Se_{61}$ film of this work exhibits a glass transition temperature of about 300-310°C which is very similar to the one reported for IG5 commercial glass of $Ge_{28}Sb_{12}Se_{60}$ composition.[2,6]

The impact of ageing on the properties of the thin film samples has been also evaluated. Indeed, since chalcogenide glasses are particularly prone to surface oxidation when exposed to air,[7] it is mandatory to control that no degradation or oxidation has occurred after several months of atmosphere exposure. All thin films show no detectable change of their optical properties after months of storage under atmosphere. Therefore, one can conclude that the 10 nm thick silicon nitride layer deposited above the chalcogenide films is an efficient protective barrier against oxidation even after more than 24 months of storage.

3. Analysis of amorphous structure of chalcogenide thin films by FTIR and Raman spectroscopies

3.1. $Ge_{1-x}Se_x$, $Ge_{1-x}S_x$ and $[Ge_{40}S_{60}]_{1-x}[Ge_{26}Se_{74}]_x$ thin films

In **Figure 2a** of the main text are shown the FTIR and Raman spectra acquired on the $Ge_{1-x}Se_x$, thin films with x varying in 0.63-0.74 range. Both FTIR and Raman spectra exhibit a monotonous evolution and refinement/hardening of vibrational modes upon increase of Se content from $Ge_{37}Se_{63}$ to $Ge_{26}Se_{74}$. Since literature on Raman modes in such glasses is more documented than IR absorption, let us first discuss Raman spectra. Raman mode centred at 195 cm^{-1} is associated to vibration of Ge-Se bond in corner-sharing (CS) $GeSe_{4/2}$ tetrahedra.[8] As the Se ratio increases, this signal dominates more and more the Raman spectra by detriment to modes centred at 179 cm^{-1} related to the Ge-Ge bonds in ethane-like (ETH) Ge_2Se_6 motifs and modes around 218 and 310 cm^{-1} corresponding to the Ge-Se bonds in edge-sharing (ES) $GeSe_{4/2}$ tetrahedra[8]. Raman signal in between these modes could correspond to several contributions. First, Se-Se bonds in Se_n chains and Se_8 rings, as found in amorphous Selenium, may appear at 234 and 252 cm^{-1}, respectively.[9] Besides, the Raman modes of Ge-Ge bonds in $Ge\text{-}Ge_mSe_{4-m}$ tetrahedral units with m = {1, 2, 3, 4} and amorphous Ge are expected at 270 and 275 cm^{-1}, respectively.[2,10] However, the main contribution at 265 cm^{-1} may be due to Se-Se bridges in between the tetrahedra as reported in $GeSe_2$ and Se-rich $Ge_{1-x}Se_x$ glasses.[11] Upon Se enrichment the increase of intensity ratio between peaks located at 195 and 218 cm^{-1} indicates an increasing number of CS $GeSe_{4/2}$ tetrahedra compared to ES $GeSe_{4/2}$ motifs accompanied by an increasing number of Se-Se bonds in short Se chains visible at 265 cm^{-1} in the amorphous $Ge_{1-x}Se_x$ network.

A similar trend is found in FTIR spectra. The shoulder of the IR absorption peak centred at 260 cm^{-1} and the two maxima near 285 and 310 cm^{-1} are getting more and more intense as the Se content increases and can be all three attributed to Ge-Se bonds.[12] Besides, at the same time

a low intense FTIR peak at 220 cm^{-1} is visible and could be assigned to ETH Ge_2Se_6 units.[12] Finally, a weak absorption band near 115 cm^{-1} may result from $GeSe_{4/2}$ tetrahedra.[12]
Our above observations are well supported by a previous study for which melt-quenched $Ge_{30}Se_{70}$ bulk glass and films were shown to consist mostly of Ge-Se-Ge bonds and in a less manner of about 15% of Ge-Se-Se-Ge bridges and 2% of Se-Se-Se chains.[5] In Ge-rich $Ge_{1-x}Se_x$ compositions, an unambiguous increase of ETH Ge_2Se_6 units upon Ge enrichment until 38 at. % of Ge has been previously reported by means of Mossbauer spectroscopy by Boolchand et al.[13] that is in excellent agreement with the trend observed here. Inversely, homopolar Ge-Ge bonds are counterintuitive for $Ge_{1-x}Se_x$ compositions with over stoichiometric Se-content (x>2). However, Ge-Ge bonds are regularly reported in Se-rich amorphous $Ge_{1-x}Se_x$ film and bulk samples.[14,15] Moreover, in case of thin film deposition and sputtering technique, atomic clusters can be sputtered from the target or condense in gas-phase when travelling from the target toward the substrate leading to possible local phase segregation at the atomic scale in the deposited films. In this work, the use of a pure Ge target during co-sputtering with $Ge_{30}Se_{70}$ in order to deposit Ge-rich $Ge_{1-x}Se_x$ films could have favoured formation of local Ge atomic clusters in the film explaining the surprising sharp increase of intensity of the Raman modes assigned to Ge-Ge bonds in Ge-enriched $Ge_{30}Se_{70}$ films. The latter observation also explains the unexpected high Ge-Ge coordination number in sputtered a-GeTe films by comparison to that expected in melt-quench amorphous GeTe.[7]

In **Figure 2b** of the main text, the FTIR spectra of $Ge_{1-x}S_x$ films exhibit one main absorption peak centred at 367 cm^{-1}. This broad peak can be decomposed as follows: the main mode at 367 cm^{-1} is due to $GeS_{4/2}$ tetrahedral motifs and its shoulder at 437 cm^{-1} denotes ES $GeS_{4/2}$ tetrahedra whereas the second shoulder at 343 cm^{-1} is related to CS $GeS_{4/2}$ tetrahedra.[16] One can also notice the presence of a broad signal centred around 147 cm^{-1} that can be attributed to Ge-S bonds.[17] Beyond these well-defined modes, the increase of IR absorption between 390 and 430 cm^{-1} can be related to numerous modes of $Ge_{1-x}S_x$ binary phase.[17]
By opposite, the Raman spectra of the $Ge_{1-x}S_x$ films are quite more complex since they result of the convolution of several modes and in particular from the contribution of the crystalline Si substrate (c-Si) at 300 and 520 cm^{-1}. The intensities of the c-Si modes increase as the S content of the film is increased. This is in part the result of a difference in films' thickness ($Ge_{40}S_{60}$ film is twice thicker than the two other films) as well as a change in the $Ge_{1-x}S_x$ chalcogenide film transparency at 532 nm due to bandgap increase with increasing the S

content (see **Optical properties of the chalcogenide thin films. Linear optical constants** section and reference[18]). Nevertheless, some features are clearly visible on the Raman spectra upon S enrichment. In a previous work, the peaks centred at 343 and 425 cm^{-1} were identified as CS $GeS_{4/2}$ tetrahedra and modes at 370 and 437 cm^{-1} assigned to ES $GeS_{4/2}$ ones.[8,17,19] The increase of the Sulphur content results in a hardening of CS $GeS_{4/2}$ mode at 343 cm^{-1} which is consistent with Raman spectra reported for GeS_2 glass.[8] Raman modes corresponding to S-S bonds in rings or chains structures (expected at 475 and 485 cm^{-1}, respectively) are not distinguishable on the Raman spectra of **Figure 2b** although they could be hidden by the shoulder of the dominant contribution at 520 cm^{-1} of c-Si substrate.[18,20] Ge-Ge modes in ethane-like units[8,13] may be at origin of Raman signal near 250 cm^{-1} and modes of $GeS_{4/2}$ tetrahedra are also visible at 115 cm^{-1}.[19] Ge-S vibration modes are expected around 150 cm^{-1} and may contribute to the broad signal visible between 100 and 180 cm^{-1}.[20,21] The Raman signal near 220 cm^{-1} increases as the S content increases but this contribution is difficult to identify. Raman intensity between 370 and 425 cm^{-1} already reported in previous works is attributed to various modes of $Ge_{1-x}S_x$ binary phases.[8,17,22-25]

In **Figure 2c** of the main text are shown the FTIR and Raman spectra acquired on the $[Ge_{40}S_{60}]_{1-x}[Ge_{26}Se_{74}]_x$ thin films obtained by co-sputtering of the $Ge_{40}S_{60}$ and $Ge_{26}Se_{74}$ targets. The FTIR and Raman spectra exhibit the main modes related to Ge-S and Ge-Se bonds described above. The relative intensities of these two main contributions depend on the $Ge_{40}S_{60}/Ge_{26}Se_{74}$ concentration ratio introduced in the film during co-sputtering.

For the $[Ge_{40}S_{60}]_{1-x}[Ge_{26}Se_{74}]_x$ films composed of both sulphide and selenide phases, all Raman and FTIR peaks exhibit a slight frequency shift of their intensity maxima compared with those of pure amorphous $Ge_{40}S_{60}$ and $Ge_{26}Se_{74}$ compounds indicating a good mixing and a limited phase separation of both phases in the sputtered films. Both Raman and FTIR modes also broaden upon mixing of the $GeSe_2$ and GeS_2 phases in $[Ge_{40}S_{60}]_{1-x}[Ge_{26}Se_{74}]_x$ films.[26] Besides, in these films a new broad Raman signal between 220 and 250 cm^{-1} appears and can be assigned to Ge-Se bonds in mixed tetrahedral $GeS_{4-m}Se_m$ structures with m = 1, 2 and 3 respectively at 265, 232 and 218 cm^{-1}, as proposed in references.[18,27] This random incorporation of sulphur and selenium in mixed $GeS_{4-m}Se_m$ tetrahedra is well supported by previous experimental and simulation studies.[28,29] Vibrations of Ge-S bonds in the $GeS_{4-m}Se_m$ mixed tetrahedral units with m = 1, 2 and 3 were simulated and found at 367, 383 and 392 cm^{-1}, respectively.[29] Note that for some of the $[Ge_{40}S_{60}]_{1-x}[Ge_{26}Se_{74}]_x$ films, a small sharp peak at 300 cm^{-1} assigned to c-Si

substrate is visible on the Raman spectra.[30] This contribution is correlated to a change of absorption of the chalcogenide layer at 2.33 eV as shown later in **Figure 5f**.

3.2. $[Ge_{30}Se_{70}]_{1-x}Sb_x$, $[Ge_{37}S_{63}]_{1-x}Sb_x$ and $[Ge_{37}S_{63}]_{1-x-y}[Ge_{30}Se_{70}]_xSb_y$ thin films

Figure 3a of main text shows the Raman and FTIR spectra acquired on the $[Ge_{30}Se_{70}]_{1-x}Sb_x$ films. In addition to phonon modes related to the $Ge_{30}Se_{70}$ amorphous phase, the FTIR and Raman spectra of Sb-doped $Ge_{30}Se_{70}$ films exhibit clear features with appearance of new peaks upon Sb addition. In the FTIR spectra, Sb addition in $Ge_{30}Se_{70}$ is accompanied by growth of a broad asymmetrical peak near 200 cm^{-1} and a shoulder on the $GeSe_2$-related peak near 250 cm^{-1} both corresponding to Sb-Se bonds vibration modes as detailed hereafter.[18,31] The progressive broadening of the peak at 200 cm^{-1} at lowest wavenumbers upon Sb enrichment can be attributed to increase of contribution of $SbSe_{3/2}$ pyramids at 180 cm^{-1} and formation of Sb-Sb bonds for instance in Se_2Sb-$SbSe_2$ ethane-like (Sb-Sb_{ETH}) motifs which are expected[18,32] at 156 cm^{-1}. We note that the IR modes of similar glasses were described as mainly the result of mixed contribution of $GeSe_{4/2}$ tetrahedral and $SbSe_{3/2}$ pyramidal structures.[31] Moreover, our observations are in good agreement with a previous study of the structure of $Ge_xSb_{40-x}Se_{60}$ (x varying from 15 to 40 at. %) glasses by means of Neutron & X-ray diffraction techniques coupled with reverse Monte Carlo simulations and IR reflectance measurements.[33] This study proposed the coexistence of $GeSe_{4/2}$ tetrahedral and $SbSe_{3/2}$ pyramidal motifs as well as the presence of Ge-Sb, Ge-Ge, Se-Se and Sb-Sb bonds depending on the Ge/Sb ratio in $Ge_xSb_{40-x}Se_{60}$ glasses. We also emphasize here that by comparison with $[GeSe_2]_{1-x}[Sb_2Se_3]_x$ films obtained by thermal co-evaporation,[32] the FTIR absorption peaks of our $[Ge_{30}Se_{70}]_{1-x}Sb_x$ thin films obtained by co-sputtering are slightly shifted in position as well as more broadened indicating a more disordered amorphous network.

In the Raman spectra of **Figure 3a**, the increase of the Sb concentration is accompanied by the appearance and growth of a broad peak centred near 160 cm^{-1} and corresponding to Sb-Sb bonds in Se_2Sb-$SbSe_2$ (Sb-Sb_{ETH}) structures which are expected[11] at 159 cm^{-1}. The high and finally dominant contribution of Raman modes near 160 cm^{-1} could be the result of a higher polarizability of Antimony bonds.[34] The decrease of the intensity ratio between peaks located at 195 and 218 cm^{-1} as the Sb content is increased indicates that CS $GeSe_{4/2}$ structures are the most impacted by Sb incorporation. One can also notice the progressive decrease of the broad band between 230 and 330 cm^{-1} upon Sb incorporation, more intense in the Raman spectrum

of $Ge_{30}Se_{70}$ film, and related to Se-Se bonds in short Se chains (265 cm^{-1}) and ES $GeSe_{4/2}$ tetrahedra (310 cm^{-1}), as discussed above. Previous works also suggested stretching vibrations of Sb-Se bonds in $SbSe_{3/2}$ structures[11] at 190 cm^{-1} but the latter are hardly discernible from the Ge-Se modes which are very close. Raman modes at 140 cm^{-1} could be also the result of Sb-Sb and Ge-Sb bonds as previously observed in amorphous Sb phase and amorphous $Ge_{15}Sb_{85}$ films deposited by sputtering.[35-37] This contribution may be responsible for asymmetrical broadening of the main Raman peak around 160 cm^{-1}. We note that the presence of Ge-Sb and Sb-Sb bonds in our thin films for high Sb content has been also evidenced elsewhere by means of an Extended X-ray Absorption Fine Structure experiment.[38] This is of a major importance since homopolar Ge-Ge, Sb-Sb and wrong Ge-Sb bonds are shown to play a major role on properties of chalcogenide glasses.[38-40]

The FTIR and Raman spectra of the $[Ge_{37}S_{63}]_{1-x}Sb_x$ thin films are shown in **Figure 3b** in the main text. The main feature visible in the FTIR spectra upon increasing the Sb content is the growth of a mode around 300 cm^{-1} by detriment to the broad peak at 370 cm^{-1} related to GeS modes. The peak of Ge-S modes near 370 cm^{-1} also shifts toward lower wavenumbers. The new peak at 300 cm^{-1} can be assigned to Sb-S vibration in $SbS_{3/2}$ structure. Its broadness is partially explained since it results from the convolution of two contributions[41,42] centred at 285 and 330 cm^{-1}.

On the Raman spectra of **Figure 3b**, Raman modes related to Ge-S bonds are centred at 340 cm^{-1} and extend in between 300 and 440 cm^{-1}. The progressive shift of the position of intensity maximum from 340 to 300 cm^{-1} is due to formation of Sb-S bonds, as mentioned above, for which the Raman modes are expected at 280 and 308 cm^{-1}.[43] The most spectacular feature upon Sb addition is the appearance of intense peaks at 140 cm^{-1} and 170 cm^{-1} assigned to Sb-Sb bonds in a-Sb phase and S_2Sb-SbS_2 ethane-like (Sb-Sb_{ETH}) structures, respectively.[36,44] A shoulder is also visible at 205 cm^{-1} and is due to Ge-Sb bonds in S_3Ge-SbS_2 structures.[45] Nevertheless, the peak centred at 170 cm^{-1} increases near linearly with the Sb concentration indicating an increasing amount of Sb-Sb homopolar bonds. Such an observation is in good agreement with previous studies.[44,46] We must note that compositions of our Sb-doped $Ge_{37}S_{63}$ films are enriched in Ge compared with that of the stoichiometric GeS_2 phase. As a result, the absence of S-S modes expected around 485 cm^{-1} as well as presence of a significant amount of Ge-Sb wrong and Sb-Sb homopolar bonds is not surprising and have been also reported in similar glasses.[23,45] In a previous study in similar S-deficient bulk glasses, whereas modelling of the EXAFS and X-ray diffraction (XRD) data required only the presence of Ge-Sb bonds,

Raman scattering data showed the presence of Ge-Ge, Ge-Sb and Sb-Sb bonds in $SbS_{3/2}$ pyramids and distorted $SbS_{5/2}$ pyramids.[23] In another study of sputtered $Ge_{35}Sb_{12}S_{53}$ thin films by means of grazing incidence X-ray absorption spectroscopy (GIXAS), both Sb-Ge and Sb-Sb bonds contributions in a 2:1 ratio were required in order to correctly model the EXAFS data.[47] When mixing GeS_2, which is composed of a random $GeS_{4/2}$ tetrahedra network and some Ge-Ge bonds,[8] with Sb_2S_3 made of $SbS_{3/2}$ pyramids and Sb-Sb bonds,[48] the amorphous structure evolves progressively. As the Sb_2S_3 content is increased, $SbS_{3/2}$ groups are formed in between the tetrahedra until an equal amount of both tetrahedral and pyramidal units is reached. Then, upon further increase of Sb_2S_3 the bridging of pyramidal $SbS_{3/2}$ motifs leads finally to progressive vanishing of $GeS_{4/2}$ groups isolated by the $SbS_{3/2}$ groups.[43,48]

In **Figure 3c** (main text) are shown the change of the FTIR and Raman spectra of the $[Ge_{37}S_{63}]_{1-x}[Ge_{30}Se_{70}]_x$ thin films upon Sb incorporation. The $[Ge_{37}S_{63}]_{1-x}[Ge_{30}Se_{70}]_x$ thin films were obtained by means of co-sputtering of $Ge_{30}Se_{70}$ and $Ge_{37}S_{63}$ targets and with aim of keeping as far as possible the composition close to the near-stoichiometric $Ge_{33}S_{37}Se_{30}$ composition. A third target of pure Sb has been added during co-sputtering deposition to study impact of Sb content on the properties of such $[Ge_{37}S_{63}]_{1-x}[Ge_{30}Se_{70}]_x$ films. First, in the FTIR spectra a progressive position shift of the Ge-S peak from 370 cm^{-1} ($GeS_{4/2}$ tetrahedra) towards lower wavenumbers and the decrease of the Ge-Se main peak near 260 cm^{-1} ($GeSe_{4/2}$ tetrahedra) is concomitant to appearance and growth of Sb-related modes near 285 cm^{-1} (Sb-S bonds in $SbS_{3/2}$ pyramids), 200 cm^{-1} (Sb-Se bonds in $SbSe_{3/2}$ pyramids) and 156 cm^{-1} (Sb in Se_2Sb-$SbSe_2$ ethane-like structure, Sb-Sb_{ETH}). The latter modes indicate that Sb element is well incorporated in the $[Ge_{37}S_{63}]_{1-x}[Ge_{30}Se_{70}]_x$ network and bonds to both S and Se chalcogen elements.

The impact of Sb on the $[Ge_{37}S_{63}]_{1-x}[Ge_{30}Se_{70}]_x$ amorphous structure is more evidenced on Raman spectra. Indeed, upon addition of the lowest concentration of Sb (~11 at. %) in $[Ge_{37}S_{63}]_{1-x}[Ge_{30}Se_{70}]_x$ one can see a dramatic decrease of the intensity of Raman modes located at 218, 232 and 265 cm^{-1} previously assigned to Ge-Se vibration in mixed $GeS_{4-m}Se_m$ tetrahedral units with m = 3, 2 and 1, respectively. Broad peaks at 367, 383 and 392 cm^{-1}, assigned to Ge-S in these mixed $GeS_{4-m}Se_m$ tetrahedral units (with m = 1, 2 and 3), decrease as well. These decrease are concomitant to reappearance of modes near 200 cm^{-1} very close to previously described CS $GeSe_{4/2}$ tetrahedra at 195 cm^{-1} as well as Sb-Se modes expected at 190 cm^{-1} (**Figure 3c** right). Vanishing of the modes of mixed $GeS_{4-m}Se_m$ tetrahedral units in favour of at least $GeSe_{4/2}$ tetrahedra indicates differences in bonding affinity of Ge/Sb atoms

depending on the S and Se chalcogen element concentration. In case of a chalcogen excess, Ge-S, Se-S and Se-Se are favoured whereas in the opposite case Ge-Se and Sb-Se are formed. Moreover, a main Raman mode at 160 cm^{-1} grows upon Sb-enrichment and is related to Sb-Sb homopolar bonds expected at 159 cm^{-1} in selenides and 170 cm^{-1} in sulphides. A previous study showed that Sb-Sb homopolar bonds form preferentially in Se-rich compositions compared with S-rich ones in the $Ge_{28}Sb_{12}S_{60-x}Se_x$ system.[46] This also tends to indicate that Ge-Se bonds are more abundant than Sb-Se, Ge-S or Sb-S ones. This observation is in agreement with our above results. By consequence, Raman signal reappearing near 200 cm^{-1} in $Ge_{30}Sb_{11}S_{33}Se_{26}$ film may result mainly from resurgence of $GeSe_{4/2}$ tetrahedra. Nevertheless, previous studies reported that Ge atoms preferentially bond with S rather than Se atoms in $Ge_{15}Sb_{20}S_{65-x}Se_x$[22] and $Ge_{23}Sb_7S_{70-x}Se_x$[18] glasses. However, these glasses are over stoichiometric in chalcogen element. The excess of S/Se element may be at origin of the observed opposite behaviour compared with our films. This could results from the promotion of Se-Se and especially Se-S bonds over Se-Sb ones allowing Ge atoms to bond with S.

3.3. $[Ge_{30}Se_{70}]_{1-x}[Ge_{52}Te_{48}]_x$, $[Ge_{30}Se_{70}]_{1-x-y}[Ge_{52}Te_{48}]_xSb_y$ and $[Ge_{1-2x}Se_xTe_x]_{1-y}Sb_y$ thin films

In the main text, **Figure 4a** shows the FTIR and Raman spectra acquired on the $[Ge_{30}Se_{70}]_{1-x}[Ge_{52}Te_{48}]_x$ thin films obtained by co-sputtering of $Ge_{30}Se_{70}$ and $Ge_{52}Te_{48}$ targets. On the FTIR spectra, as the $Ge_{52}Te_{48}$ content is increased the Ge-Se main modes centred at 260 and 309 cm^{-1} vanish with simultaneously the growth of a shoulder at wavenumbers below 260 cm^{-1}. This shoulder can be attributed to GeTe modes since the FTIR absorption main peaks of a-GeTe are expected[49] at 150 and 220 cm^{-1}. A small shift of the Ge-Se main peak towards lower wavenumbers could also indicate a possible effect of the presence of a heavier chemical element such as Te in Ge-Se environment.

In the Raman scattering signal of the $[Ge_{30}Se_{70}]_{1-x}[Ge_{52}Te_{48}]_x$ thin films, the contribution of CS $GeSe_{4/2}$ tetrahedra centred at 195 cm^{-1} progressively vanishes as the $Ge_{52}Te_{48}$ content is increased. More interestingly, upon introduction of $Ge_{52}Te_{48}$ a new peak appears at 180 cm^{-1} that is progressively broadening as its position shifts from 180 to 145 cm^{-1}. This broad peak can be assigned to Ge-Te bonds in $GeTe_{4-n}Se_n$ (n = {0, 1, 2, 3}) structures for which n decreases as the $Ge_{52}Te_{48}$ content is increased. Indeed, the most intense Raman peak of a-GeTe phase, which is related to both $Ge\text{-}GeTe_3$ tetrahedra or GeTe defective octahedral motifs of a-

GeTe[50,51], is expected near 120 cm^{-1}.[34] The broadening of this new peak concomitant to $Ge_{52}Te_{48}$ addition is the result of an increase of disorder and the presence of highly controverted Te-Te homopolar bonds which were reported to appear at 150 cm^{-1}.[52] In Te-deficient $Ge_{1-x-y}Sb_xTe_y$ glasses, no Te-Te homopolar bonds were found.[53] However, some reports evidenced that Te-rich Ge_xTe_{1-x} glasses can exhibit Te-Te bonds with Raman contribution near 150 cm^{-1} as well as at 157 cm^{-1} in the amorphous Te phase.[52,54,55] Incorporation of Se-rich $Ge_{1-x}Se_x$ may increase segregation of a Te-rich phase. Therefore, Te-Te homopolars cannot be excluded in our samples but drawing a definitive conclusion is difficult in view of the data. We cannot also exclude Se-Te bonds which are expected to appear near 200 cm^{-1}.[56] Indeed, in a previous work[5] Te-Se bonds were proposed in Ge-Se-Te-Se-Ge motifs due to the reduction of oxidation state of Ge to +II resulting from the substitution of Se atoms by Te in the $Ge_{30}Se_{70-x}Te_x$ glass for x up to 20. Furthermore, concerning the presence of homopolar bonds, the Raman peak appearing at ~170 cm^{-1} upon $Ge_{52}Te_{48}$ incorporation in $Ge_{30}Se_{70}$ could be due to Ge-Ge homopolar bonds in Se_3Ge-$GeSe_3$ ETH structures. Both shift and broadening of the latter contribution towards the lower wavenumbers could be attributed to Ge-Ge bonds getting more and more located in a Te-rich environment, such as in Ge-$GeTe_{3-n}Se_n$ motifs with n decreasing and reminiscent to the abovementioned $GeTe_{4-n}Se_n$ motifs.[52] Upon increase of the $Ge_{52}Te_{48}$ concentration, despite the decrease of the Raman intensity at 310 cm^{-1} attributed to ES $GeSe_{4/2}$ tetrahedra, the Raman intensity around 276 cm^{-1} remains almost constant suggesting again the presence in the GeTe-rich $[Ge_{30}Se_{70}]_{1-x}[Ge_{52}Te_{48}]_x$ films of Ge-Ge homopolars in $GeTe_{4-n}Ge_n$ motifs.[34]

In **Figures 4b,c** of the main text are shown the FTIR and Raman spectra of $[Ge_{30}Se_{70}]_{1-x-y}[Ge_{52}Te_{48}]_xSb_y$ and $[Ge_{1-2x}Se_xTe_x]_{1-y}Sb_y$ thin films obtained by co-sputtering of $Ge_{30}Se_{70}$, $Ge_{52}Te_{48}$ and Sb targets. The plotted spectra are divided into two series of sample: a first one of $[Ge_{30}Se_{70}]_{1-x-y}[Ge_{52}Te_{48}]_xSb_y$ thin films for which the Ge content was kept almost constant but the (Sb+Te)/Se ratio is progressively increased (**Fig. 4b**) and a second set of thin film samples ($[Ge_{1-2x}Se_xTe_x]_{1-y}Sb_y$) for which only the Sb content was increased in a host compound fixed to $Ge_{43}Se_{29}Te_{28}$ (**Fig. 4c**). The comparison between **Figures 4b** and **4c** is thus instructive since it gives insights on how a comparable Sb concentration (~15, 20 and 25 at %) affects the structure of two chalcogenide matrices differing by their Ge/Se/Te atomic ratio.

First, a general observation arises from comparison of the FTIR spectra of **Figures 4b,c**. The broad absorption signal below 200 cm^{-1} evolves in a different manner depending on the sample's composition. In thin films containing both Sb and Te, a broad peak around 150 cm^{-1} is visible whereas the latter is not visible nor intense in FTIR spectra of $[Ge_{30}Se_{70}]_{1-x}[Ge_{52}Te_{48}]_x$

and $[Ge_{30}Se_{70}]_{1-x}Sb_x$ films. This broad peak can be reasonably attributed to convolution of contributions of the modes of Se_nTe_{2-n}-Sb-Sb-Se_nTe_{2-n} (with n =1 or 2 depending on the Se/Te ratio) mixed ethane-like structure (Sb-Sb_{ETH}) and in a less extent those corresponding to a-GeTe phase expected at 156 cm^{-1} and 150 cm^{-1}, respectively. Besides, by a comparison between films exhibiting a same Sb level in **Figure 4b,c**, one can note that an increase of the $[Ge_{52}Te_{48}]/[Ge_{30}Se_{70}]$ ratio provokes a decrease of absorption peaks related to Ge-Se and Sb-Se bonds with maxima centred respectively at 260 and 200/250 cm^{-1} but with an increase of the broad absorption band between 100 and 250 cm^{-1}. The latter trend in the [100-250] cm^{-1} range can be related to an increase of the contribution of GeTe-related modes such as Ge-Te bonds in Ge-$GeTe_3$ tetrahedra and GeTe defective octahedra (broad peaks centred at 150 and 220 cm^{-1})[51] as well as contribution of Sb-Sb bonds in Sb-Sb_{ETH} motifs (156 cm^{-1}). At the same time, the increase of the Te content upon increase of the $[Ge_{52}Te_{48}]/[Ge_{30}Se_{70}]$ ratio is accompanied by a shift to lower wavenumbers and broadening of $GeSe_{4/2}$ tetrahedra main contribution at 260 cm^{-1}.

From analysis of the Raman spectra of **Figures 4b,c**, similar conclusions can be drawn. In **Figure 4b**, the impact of Sb/Te introduction by detriment to the Se content on the $[Ge_{30}Se_{70}]_{1-x}Sb_x$ films' structure is showing a double effect. First, the Raman mode corresponding to the CS $GeSe_{4/2}$ tetrahedra at 195 cm^{-1} progressively vanishes in favour of a peak near 160 cm^{-1} which increases and shifts slightly towards lower wavenumbers. This is probably due to the growth of Sb-Te bonds contribution expected[53] at 145 cm^{-1} as well as in a less extent contribution of Ge-Te modes at 120 cm^{-1} for compositions with the highest $Ge_{52}Te_{48}$ contents. The presence of Te-Te bonds at 150 cm^{-1} cannot be excluded even if Ge-Te related modes are also clearly evidenced by FTIR. Secondly, the impact of the progressive Se substitution by Sb/Te in $[Ge_{30}Se_{70}]_{1-x}Sb_x$ films is accompanied by a decrease of the broad Raman contribution in the [250-325] cm^{-1} range. The decrease of this band can be attributed mainly to the decrease of ES $GeSe_{4/2}$ tetrahedra in favour of Sb-Sb and Ge-Te modes.

The impact of Sb introduction in $Ge_{1-2x}Se_xTe_x$ films on the Raman spectra of **Figure 4c** helps to identify the origin of the broad Raman contribution at ~150 cm^{-1}. As shown in **Figure 4a** for $[Ge_{30}Se_{70}]_{1-x}[Ge_{52}Te_{48}]_x$ films, the peak near 150 cm^{-1} is mainly due to modes of isolated Ge-$GeTe_3$ tetrahedra and GeTe defective octahedra in a Se environment due to the slight shift towards higher wavenumbers compared to modes in the pure a-GeTe phase expected at 120 cm^{-1}. Then, upon Sb addition in $Ge_{1-2x}Se_xTe_x$ films, one can observe the splitting of the above-mentioned mode at ~150 cm^{-1} into two convoluted contributions which can be described as

follows. A first mode is due to the Raman peak near 145 cm^{-1} related to the Ge-Te bonds in $GeTe_{4-n}Se_n$ (n = {0, 1, 2, 3}) structures. A second convoluted mode at 159 cm^{-1}, attributed to Sb-Sb bonds in Se_nTe_{2-n}-Sb-Sb-Se_nTe_{2-n} structures (Sb-Sb_{ETH}), grows as the Sb concentration is increased. Simultaneously, the Raman signal remaining around 200 cm^{-1} can reasonably correspond to Sb-Se bonds as observed in FTIR spectra. The latter increases with the Sb content by detriment to Ge-Se (CS $GeSe_{4/2}$ tetrahedra), Ge-Ge or Se-Se bonds as evidenced by the concomitant decrease of the signal in the 250-300 cm^{-1} range. However, in these Raman spectra, due to their close positions, distinguishing Sb-Sb from Sb-Te and even Te-Te contribution near 150 cm^{-1} remains difficult.

4. Modelling of the spectroscopic ellipsometry data and the Cody-Lorentz model

The Cody-Lorentz model is an extension of the well-known Tauc-Lorentz model. The TL model is well adapted to amorphous semiconductors but the CL includes an additional term in order to better describe the Urbach absorption.[57,58] Therefore, the CL model allows to accurately model the optical behaviour of chalcogenide glasses in terms of transparency and interband absorption regions (**Figure S1**). Amorphous chalcogenide band gap absorption region is complex to reproduce mathematically and combination of several different models have been used in previous studies in order to achieve satisfying fits in this transparency window extremum.[59] The present study does not pretend to perfectly describe the dielectric constants changes at high energy. The main objective is to obtain the trend on the complex refractive index in transparency region depending on films' compositions. In this spectral range, a single CL model is well adapted to fit ellipsometry data with a good accuracy and limited error (see **Figure S1**). However, using this CL model we note a lower fitting accuracy when the band gap absorption region of the chalcogenide films increases toward middle of the energy range of measurement (400-1700 nm or 0.73-3.1 eV). Counterintuitively, in case of Sb or $Ge_{52}Te_{48}$ doped $Ge_{30}Se_{70}$ films the fitting accuracy is very high and increases with the Sb or $Ge_{52}Te_{48}$ content. By comparison, in case of a-$Ge_{30}Se_{70}$ thin film, the modelling accuracy is slightly lower. We attributed this result to contribution of in gap defect states and band tails introduced by homopolar Se-Se bonds. Such electronic states are not taken into account in the Cody-Lorentz formula. By opposite, in case of Sb or $Ge_{52}Te_{48}$ addition in a-$Ge_{30}Se_{70}$ films, the amount of chalcogen elements in excess from the stoichiometric compounds ($GeSe_2$, GeTe, Sb_2Se_3) decreasing, formation of Se-Se or Te-Te homopolars is thus limited explaining the better fitting accuracy. Nevertheless, the creation of electronic defect states under the

conduction band (mid-gap states) or on top of the valence band (band tails) due to Ge-Ge, Sb-Sb homopolar and Ge-Sb wrong bonds might be also taken into account.[60] Finally, the CL modelling of $Ge_{30}Se_{70}$ film gives values which are highly consistent with previous literature in particular at 1550nm.[3]

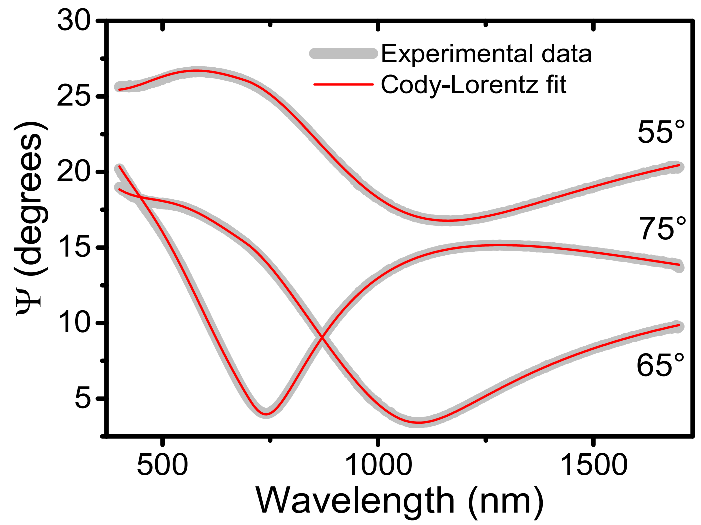

Figure S1. Cody-Lorentz modelling of $Ge_{26}Sb_{22}S_{29}Se_{23}$ thin film deposited by co-sputtering.

5. n_2 nonlinear refractive indices determination by means of the Sheik-Bahae model

In a previous work it has been shown that the Sheik-Bahae model systematically overestimates Kerr indices.[61] In the present work, we emphasize that the band gap values we used in the Sheik-Bahae model were deduced by means of the E_g^{04} method. This method gives band gap energy values higher than that obtained by means of other methods such as for instance Tauc's plots. Therefore, using such overestimated E_g^{04} band gap values as input in the Sheik-Bahae formula may have corrected somehow the drawback of this model since the band gap energy value inversely contributes to 3rd order non-linear coefficient. Besides, other previous studies emphasized the accuracy limit of such models to calculate the Kerr index n_2 of glasses near the fundamental absorption band edge.[62] An absorption of 1 cm^{-1} is invoked as being a limit value allowing to discriminate weak absorption from the Urbach absorption. Thus, based on the latter assumption using the Sheik-Bahae or Dinu models would indicate that for the vast majority of our films non-realistic estimation of n_2 could be inaccurate at 1550 nm wavelength. Some discrepancy between experimental and calculated values were observed when also considering TPA. Indeed, TPA becomes non-negligible below $E_g/2$ in amorphous

materials, as a result of possible transitions from defect states at energy levels located in the band gap of the amorphous material.

Besides, the n_2 values determined by Z-scan technique are more consistent with those calculated by means of the Dinu's model compared with the Sheik-Bahae one.[63] This discrepancy between both models is explained by the fact that, by opposite to the Sheik-Bahae model which was developed for direct band gap materials, the Dinu's formula is more accurate to describe the behaviour of indirect band gap materials as well as in order to take into account phonon transitions. Both models were initially based on theory of crystalline semiconductors. Dinu's model predicts a maximum for n_2 at an energy of 0.65 x E_g^{opt} so slightly higher than the one in the Sheik-Bahae formula.

References

1. Wang, H. *et al.* Compositional dependence of crystallization and structural stability in Ge-Sb-Se chalcogenide films. *J. Non-Cryst. Solids* **453**, 108-112 (2016).
2. Olivier, M. *et al.* Photosensitivity of pulsed laser deposited Ge-Sb-Se thin films. *Opt. Mater. Express* **5**, 781-793 (2015).
3. Raty, J.-Y. *et al.* Aging mechanisms in amorphous phase-change materials. *Nat. Commun.* **6**, 7467 (2015).
4. Yang, G. *et al.* Physical properties of the Ge_xSe_{1-x} glasses in the $0<x<0.42$ range in correlation with their structure. *J. Non-Cryst. Solids* **377**, 54-59 (2013).
5. Suriñach, S. Baró, M. D. Clavaguera-Mora, M. T. & Clavaguera, N. Glass forming ability and cristallization kinetics of allows in the $GeSe_2$-GeTe-Sb_2Te_3 system. *J. Non-Cryst. Solids* **111**, 113-119 (1989).
6. Klocek, P. & Colombo, L. Index of refraction, dispersion, bandgap and light scattering in GeSe and GeSbSe glasses. *J. Non-Cryst. Solids* **93**, 1-16 (1987).
7. Noé, P. *et al.* Impact of interfaces on scenario of crystallization of phase change materials. *Acta Mater.* **110**, 142–148 (2016).
8. Jackson, K. *et al.* Raman-active modes of a-$GeSe_2$ and a-GeS_2: A first-principles study. *Phys. Rev. B* **60**, 14985-14989 (1999).
9. Sugai, S. Stochastic random network model in Ge and Si chalcogenide glasses. *Phys. Review B* **35**, 1345-1361 (1987).

10. Lannin, J. S. Maley N. & Kshirsagar, S. T. Raman scattering and short range order in amorphous germanium. *Solid State Commun.* **53**, 939-942 (1985).
11. Halenkovič, T. *et al.* Amorphous Ge-Sb-Se thin films fabricated by co-sputtering: Properties and photosensitivity. *J. Am. Ceram. Soc.* **101**, 1-13 (2018).
12. Fukunaga, T. Tanaka, Y. & Murase, K. Glass formation and vibration properties in the (Ge,Sn)-Se system. *Solid State Commun.* **42**, 513-516 (1982).
13. Boolchand, P. Grothaus, J. Tenhover, M. Hazle, M. A. & Grasselli, R. K. Structure of GeS2 glass: Spectroscopic evidence for broken chemical order. *Phys. Rev. B* **33**, 5421 (1986).
14. Pan, R.K. *et al.* Structure and optical properties of amorphous Ge–Se films prepared by pulsed laser deposition. *Optik* **124**, 4943– 4946 (2013).
15. Zhang, S. Chen, Y. Wang, R. Shen, X. & Dai, S. Observation of photobleaching in Ge-defiencient $Ge_{16.8}Se_{83.2}$ chalcogenide thin film with prolonged irradiation. *Sci. Rep.* **7**, 14585 (2017).
16. Lucovsky, G. Nemanich, R. J. & Solin, S. A. Coordination dependent vibrational properties of amorphous semiconductor alloys. *Solid State Commun.* **17**, 1567-1572 (1975).
17. Julien, C. *et al.* Raman and infrared spectroscopic studies of Ge-Ga-Ag sulphide glasses. *Mat. Sci. Eng. B* **22**, 191-200 (1994).
18. Petit, L. *et al.* Effect of the substitution of S for Se on the structure of the glasses in the system $Ge_{0.23}Sb_{0.07}S_{0.70-x}Se_x$. *J. Phys. Chem. Solids* **66**, 1788–1794 (2005).
19. Tao, H. Mao, S. Dong, G. Xiao, H. & Zhao, X. Raman scattering studies of the Ge-In sulphide glasses. *Solid State Commun.* **137**, 408–412 (2006).
20. Guo, H.T. Zhang, M.-J., Xu, Y.-T. Xia, X.-S. & Yang, Z.-Y. Structural evolution study of additions of Sb_2S_3 and CdS into GeS_2 chalcogenide glass by Raman spectroscopy. *Chin. Phys. B* **26**, 104208 (2017).
21. Lucovsky, G. Galeener, F. L. Keezer, R. C. Geils, R. H. & Six, H. A. Structural interpretation of the infrared and Raman spectra of glasses in the alloy system $Ge_{1-x}S_x$. *Phys. Review B* **10**, 5134-5146 (1974).
22. Wang, R. *et al,* Structure and physical properties of $Ge_{15}Sb_{20}Se_{65-x}S_x$ glasses *J. Am. Ceram. Soc.* **101**, 201-207 (2018).
23. Pethes, I. *et al.* Atomic level structure of Ge-Sb-S glasses: Chemical short range order and long Sb-S bonds. *J. Alloy. Compd.* **774**, 1009-1016 (2019).

24. Frumarová, B. Němec, P. Frumar, M. Oswald, J. & Vlček, M. Synthesis and optical properties of the Ge-Sb-S:$PrCl_3$ glass system. *J. Non-Cryst. Solids* **256&257** 266–270 (1999).
25. Petit, L. *et al.* Correlation between physical, optical and structural properties of sulfide glasses in the system Ge-Sb-S. *Mat. Chem. Phys.* **97** , 64-70 (2006).
26. Gu, S. Zhang, Q. Pan, R. Microstructure and mid-infrared refracive index dispersion of $Ge(S_xSe_{1-x})_4$ glasses. Chalcogenide Lett. 12, 257-262 (2015).
27. Griffiths, J. E. Espinosa, G. P. Phillips, J. C. & Remeika, J. P. Raman spectra and athermal laser annealing of $Ge(S_xSe_{1-x})_2$ glasses. *Phys. Rev. B* **28**, 4444–4453 (1983).
28. Dongol, M. Elhady, A. F. Ebied, M. S. & Abuelwafa, A. A. Impact of sulfur content on structural and optical properties of $Ge_{20}Se_{80-x}S_x$ chalcogenide glasses thin films. *Opt. Mat.* **78**, 266-272 (2018).
29. Xuecai, H. Guangying, S. Yu, L. Hongbo, Y. & Yonghua, L. Structure and vibrational modes of Ge-S-Se glasses : Raman scattering and ab initio calculations. *Chalcogenide Lett.* **9**, 465-474 (2012).
30. Hu, J. et al. Exploration of waveguide fabrication from thermally evaporated Ge–Sb–S glass films, Opt. Mater. 30, 1560-1566 (2008).
31. Quiroga, I. et al. Infrared studies of a $Ge_{0.20}Sb_{0.05}Se_{0.75}$ glassy semiconductor. *J. Non-Cryst. Solids* **196**, 183-186 (1996).
32. Petkov, K. Vassilev, G. Todorov, R. Tasseva, J. & Vassilev, V. Optical properties and structure of thin films from the system $GeSe_2$–Sb_2Se_3–AgI. *J. Non-Cryst. Solids* **357**, 2669–2674 (2011).
33. Fabian, M. Dulgheru, N. Antonova, K. Szekeres, A. & Gartner, M. Investigation of the atomic structure of Ge-Sb-Se chalcogenide glasses. *Adv. Condens. Matter Phys.* **2018**, 1–11 (2018).
34. Andrikopoulos, K. S. Yannopoulos, S. N. Kolobov, a. V. Fons, P. & Tominaga, J. Raman scattering study of GeTe and $Ge_2Sb_2Te_5$ phase-change materials. *J. Phys. Chem. Solids* **68**, 1074–1078 (2007).
35. Lannin, J. S. Raman scattering properties of amorphous As and Sb. *Phys. Rev. B* **15**, 3863-3871 (1977).
36. Rossow, U. *et al.* Growth mode of ultrathin Sb layers on Si studied by spectroscopic ellipsometry and Raman scattering. *Appl. Surf. Sci.* **63**, 35-39 (1993).

37. Shakhvorostov, D. *et al.* Evidence for electronic gap-driven metal-semiconductor transition in phase-change materials. *PNAS* **106**, 10907-10911 (2009).
38. Noé, P. *et al.* Toward ultimate nonvolatile resistive memories: The mechanism behind ovonic threshold switching revealed. *Science Advances* **6**, eaay2830 (2020).
39. Lee, J. H. *et al.* Unravelling interrelations between chemical composition and refractive index dispersion of infrared-transmitting chalcogenide glasses. *Sci. Rep.* **8**, 15482 (2018).
40. Verdy, A. *et al.* Improved electrical performance thanks to Sb and N doping in Se-rich GeSe-Based OTS selector devices. *IEEE 9th Int. Mem. Work. , IMW* (2017).
41. Droichi, M. S.Vaillant, F.Bustarret, E. & Jousse, D. Study of localized states in amorphous chalcogenide Sb_2S_3 films. *J. Non-Cryst. Solids* **101**, 151-155 (1988).
42. Kamitsos, E. I. Kapoutsis, J. A. Culeac, I. P. & Iovu, M. S. Structure and bonding in As-Sb-S chalcogenide glasses by infrared reflectance spectroscopy. *J. Phys. Chem. B* **101**, 11061–11067 (1997).
43. Lin, C. *et al.* Network structure in GeS_2-Sb_2S_3 Chalcogenide glasses: Raman spectroscopy and phase transformation study. *J. Phys. Chem. C* **116**, 5862–5867 (2012).
44. Watanabe, I. *et al.* Study on local structure in amorphous Sb-S films by Raman scattering. *J. Non-Cryst. Solids* **58**, 35–40 (1983).
45. Nazabal, V. Carpentier, F. & Adam, J.-L. *Int.* Sputtering and pulsed laser deposition for near- and mid-infrared applications: A comparative study of $Ge_{25}Sb_{10}S_{65}$ and $Ge_{25}Sb_{10}Se_{65}$ amorphous thin films. *J. Appl. Ceram. Technol.* **8**, 990–1000 (2011).
46. Guery, G. *et al.* Evolution of glass properties during a substitution of S by Se in $Ge_{28}Sb_{12}S_{60-x}Se_x$ glass network. *J. Non-Cryst. Solids* **358**, 1740-1745 (2012).
47. d'Acapito, F. *et al.* Role of Sb dopant in Ag:GeS_x-based conducting bridge random access memories. *Phys. Status Solidi A* **213**, 311-315 (2016).
48. Svoboda, R. Málek, J. & Liška, M. Correlation between the structure and relaxation dynamics of $(GeS_2)_y(Sb_2S_3)_{1-y}$ glassy matrices. *J. Non-Cryst. Solids* **479**, 113-119 (2018).
49. Raty, J.-Y. *et al.* Vibrational properties and stabilization mechanism of the amorphous phase of doped GeTe. *Phys. Rev. B* **88**, 014203 (2013).

50. Upadhyay, M. Murugavel, S. Anbarasu, M. & Ravindran, T. R. Structural study on amorphous and crystalline state of phase change material. *J. Appl. Phys.* **110**, 083711 (2011).
51. Raty, J.-Y. Aging in phase change materials: Getting insight from simulation. *Phys. status solidi - Rapid Res. Lett.* **1800590**, 1800590 (2019).
52. Varma, G. S. *et al.* Thermally reversing window in $Ge_{15}Te_{85-x}In_x$ glasses : Nanoindentation and micro-Raman studies. *J. Non-Cryst. Solids* **358**, 3103–3108 (2012).
53. Němec, P. *et al.* Amorphous and crystallized Ge-Sb-Te thin films deposited by pulsed laser: Local structure using Raman scattering spectroscopy. *Mater. Chem. Phys.* **136**, 935-941 (2012).
54. Uemura, O. Hayasaka, N. Tokairin, S. & Usuki, T. Local atomic arrangement in Ge-Te and Ge-S-Te glasses. *J. Non. Cryst. Solids* **205–207**, 189–193 (1996).
55. Brodsky, M. H. Gambino, R. J. Smith, J. E. & Yacoby, Y. The Raman spectrum of amorphous tellurium. *Phys. Stat. sol. B* **52**, 609 (1972).
56. Mendoza-Galvan, A. García-García, E. Vorobiev, Y. V. & González-Hernández, J. Structural, optical and electrical characterization of amorphous Se_xTe_{1-x} thin film alloys. *Microelectron. Eng.* **51-52**, 677-687 (2000).
57. Němec, P. Přikryl, J. Nazabal, V. & Frumar, M. Optical characteristics of pulsed laser deposited Ge-Sb-Te thin films studied by spectroscopic ellipsometry. *J. Appl. Phys.* **109**, 073520 (2011).
58. Orava, J. *et al.* Optical properties and phase-change transition in $Ge_2Sb_2Te_5$ flash evaporated thin films studied by temperature dependent spectroscopic ellipsometry. *J. Appl. Phys.* **104**, 043523 (2008).
59. Abdel-Wahab, F. *et al.* Spectroscopic ellipsometry characterization of $Ge_{30-x}Sb_x$ Se_{70} films using combinations of multiple dispersion functions. *Optik* **147**, 59–71 (2017).
60. Černošek, Z. Černošková, E. Hejdová, M. Holubová, J. Todorov, R. *et al.* The properties and structure of Ge-Se-Te glasses and thin film. *J. Non-Cryst. Solids* **460** 169–177 (2017).
61. Boudebs, G. Sanchez, F. Troles, J. & Smektala, F. Nonlinear optical properties of chalcogenide glasses: Comparison between Mach-Zehnder interferometry and Z-scan techniques. *Optics Comm.* **199**, 425-433 (2001).

62. Romanova, E. *et al.* Measurement of non-linear optical coefficients of chalcogenide glasses near the fundamental absorption band edge. *J. Non-Cryst. Solids* **480**, 13-17 (2018).
63. Dinu, M. Dispersion of phonon-assisted nonresonant third-order nonlinearities. *IEEE J. Quantum Electron.* **39**, 1498–1503 (2003).
